# MMP activation–associated aminopeptidase N reveals a bivalent 14-3-3 binding motif

**DOI:** 10.1074/jbc.RA120.014708

**Published:** 2021-01-13

**Authors:** Sebastian Kiehstaller, Christian Ottmann, Sven Hennig

**Affiliations:** 1 Department of Chemistry and Pharmaceutical Sciences, VU University Amsterdam, Amsterdam, Netherlands; 2 Amsterdam Institute of Molecular and Life Sciences (AIMMS), VU University Amsterdam, Amsterdam, Netherlands; 3 Laboratory of Chemical Biology, Department of Biomedical Engineering and Institute for Complex Molecular Systems, Eindhoven University of Technology, Eindhoven, Netherlands

**Keywords:** aminopeptidase N, CD13, extracellular 14-3-3, crystal structure, protein-protein interaction, aminopeptidase, 14-3-3 protein, X-ray crystallography, structural biology

## Abstract

Aminopeptidase N (APN, CD13) is a transmembrane ectopeptidase involved in many crucial cellular functions. Besides its role as a peptidase, APN also mediates signal transduction and is involved in the activation of matrix metalloproteinases (MMPs). MMPs function in tissue remodeling within the extracellular space and are therefore involved in many human diseases, such as fibrosis, rheumatoid arthritis, tumor angiogenesis, and metastasis, as well as viral infections. However, the exact mechanism that leads to APN-driven MMP activation is unclear. It was previously shown that extracellular 14-3-3 adapter proteins bind to APN and thereby induce the transcription of MMPs. As a first step, we sought to identify potential 14-3-3–binding sites in the APN sequence. We constructed a set of phosphorylated peptides derived from APN to probe for interactions. We identified and characterized a canonical 14-3-3–binding site (*site 1*) within the flexible, structurally unresolved N-terminal APN region using direct binding fluorescence polarization assays and thermodynamic analysis. In addition, we identified a secondary, noncanonical binding site (*site 2*), which enhances the binding affinity in combination with *site 1* by many orders of magnitude. Finally, we solved crystal structures of 14-3-3σ bound to mono- and bis-phosphorylated APN-derived peptides, which revealed atomic details of the binding mode of mono- and bivalent 14-3-3 interactions. Therefore, our findings shed some light on the first steps of APN-mediated MMP activation and open the field for further investigation of this important signaling pathway.

Aminopeptidase N (APN, CD13) is a zinc-dependent ectopeptidase of the M1 family. It is a type II integral membrane protein and is located on the surface of many mammalian cells like fibroblasts, epithelial and myeloid cells (1, 2). APN consists of 967 amino acids (aa), which can be divided into three regions. A short N-terminal region is located in the cytoplasm (aa 1–9), followed by a single-helix transmembrane domain (aa 10–27) and a large extracellular region (aa 28–967) (3). APN is involved in multiple processes. It is most widely known for its protease activity in the renin-angiotensin system, where it proteolytically converts angiotensin III to IV (4). In addition to its enzymatic activity, it functions as a receptor for coronaviruses and has been proposed to participate in the endocytosis of cholesterol (5, 6, 7). Some of the functions of APN are mediated by protein-protein interactions. Binding of extracellular 14-3-3 proteins, for instance, was shown to induce transcription of various matrix-metalloproteinases (MMPs) via p38 MAPK signaling (8, 9, 10, 11, 12). MMPs act in tissue remodeling by rearranging the extracellular matrix (13). They are involved in several human diseases, such as fibrosis and rheumatoid arthritis (14, 15). MMPs play also important roles in diverse types of cancers by promoting angiogenesis and metastasis (16, 17).

The family of 14-3-3 proteins are highly conserved eukaryotic adapter proteins, which are involved in several hundred protein-protein interactions and therefore a plethora of cellular functions. Seven homologs are present in human (β/α, η, σ, ζ, τ, ε, and γ) with a molecular mass of ∼30 kDa (18). 14-3-3 consists of nine α-helices and forms via its N-terminal dimerization region homo- and heterodimers in solution (19, 20). Each of the two protomers possesses an amphipathic binding groove to interact with their partner. 14-3-3 binding occurs usually in a phosphorylation-dependent manner, in which a serine or threonine of the target protein is phosphorylated and subsequently able to bind to a conserved basic patch within 14-3-3 (21, 22). Due to its dimeric nature, each 14-3-3 dimer harbors two of these binding grooves, and several studies have shown bivalent binding between 14-3-3 dimers and their interaction partner (23, 24, 25, 26). Throughout the last decade, despite its widespread intracellular roles, some 14-3-3 homologs (*e.g.* β/α, η, σ (also known as stratifin (SFN)), ζ, and ε) were shown to be secreted and are also present in the extracellular space. Up to now, extracellular 14-3-3 could be linked to functions, among others, in collagenase expression or rheumatoid arthritis or as an anti-fibrogenic factor (10, 14, 15, 27, 28). In contrast to the intracellular signal transduction that induces MMPs via the p38 MAPK pathway, the extracellular stimulation of APN by 14-3-3 is less understood. Extracellular 14-3-3 binds directly to APN in a phosphorylation-dependent manner (11, 29). This interaction can be suppressed by blocking the 14-3-3 binding groove either by small-molecule inhibitors (phosphonates) or peptidomimetics (30, 31). Also, an APN-derived peptide containing a phosphorylated tyrosine was shown to inhibit the 14-3-3–mediated MMP induction (29).

To identify the region of direct interaction between APN and 14-3-3, we showed the detailed analysis of potential binding epitopes. Using phosphorylated peptides, we identified, validated (direct fluorescence polarization (FP)-binding assay), and in-depth characterized (by isothermal titration calorimetry (ITC)) potential APN phosphorylated serine- and threonine-based 14-3-3–binding sites. We showed that a second noncanonical 14-3-3–binding site increases the affinity and therefore implicates a bivalent interaction mode. Additionally, we solved the crystal structure of mono- and bis-phosphorylated APN stretches in complex with 14-3-3σ. Therefore, we contribute a detailed analysis of a bivalent 14-3-3 interaction partner and highlight the importance of secondary, noncanonical 14-3-3–binding sites.

## Results

### Aminopeptidase N contains canonical 14-3-3–binding motifs

**Figure 1:**
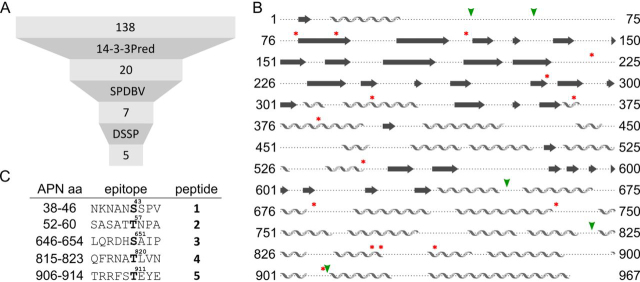
**Identification of potential 14-3-3–binding APN epitopes.***A*, workflow to identify potential 14-3-3–binding APN epitopes (14-3-3–binding prediction server (14-3-3Pred), Swiss PDB Viewer (SPDBV), and DSSP (36,37, 38, 39,40). *B*, overview of the location of the final 5 residues (*green arrowheads*) and the 15 residues filtered out by SPDBV and DSSP (*red asterisks*). *C*, resulting panel of five potential APN epitope candidates (**1–5**), including their position in APN, sequence, phosphorylation position, and peptide number.

For a detailed analysis of APN binding to 14-3-3, we aimed for the characterization of its exact binding mode. As often phosphorylated Ser and Thr residues are found in 14-3-3–binding sites (32, 33, 34, 35), we performed an analysis of all 138 extracellular Ser and Thr residues of APN using three selection criteria (Fig. 1*A*).

First, we analyzed the potential of each Ser/Thr-containing sequence motif for 14-3-3 binding (14-3-3Pred (36)). From the three 14-3-3Pred classifiers, we defined that at least one threshold needed to be met. This resulted in 20 potential 14-3-3–binding regions within APN (Table S1). Second, we reasoned that the according Ser/Thr residue should be surface accessible (≥20%, Swiss PDB Viewer (37)), which narrowed the number of candidates down to seven potential 14-3-3–binding sites. Two of these (Ser^43^ and Thr^57^) are located in the structurally nonresolved N-terminal region of APN (PDB entry 4FYQ (3)). Finally, the potential binding motifs needed to be located in accessible, flexible secondary structural elements (the define secondary structure of proteins algorithm (DSSP) via MRS, Fig. 1*B* (38, 39, 40)), which resulted in five potential 14-3-3–binding sites (Fig. 1*C*).

### Phosphorylated APN pSer^43^ binds extracellular 14-3-3

Our sequence and structural assessment of potential 14-3-3–binding motifs within APN revealed five potential candidates. To validate these motifs, we synthesized peptides **1–5** (Fig. 1*C*) via standard solid-phase peptide synthesis (SPPS). Each peptide was composed of five N-terminal (–5) and three C-terminal (+3) amino acids relative to the pSer/pThr position and an N-terminal FITC attached via a PEG2 linker. The well-established 14-3-3–binding sequence of RAF1 (RQRSTpSTPN) was used as a positive control and synthesized similarly.

**Figure 2:**
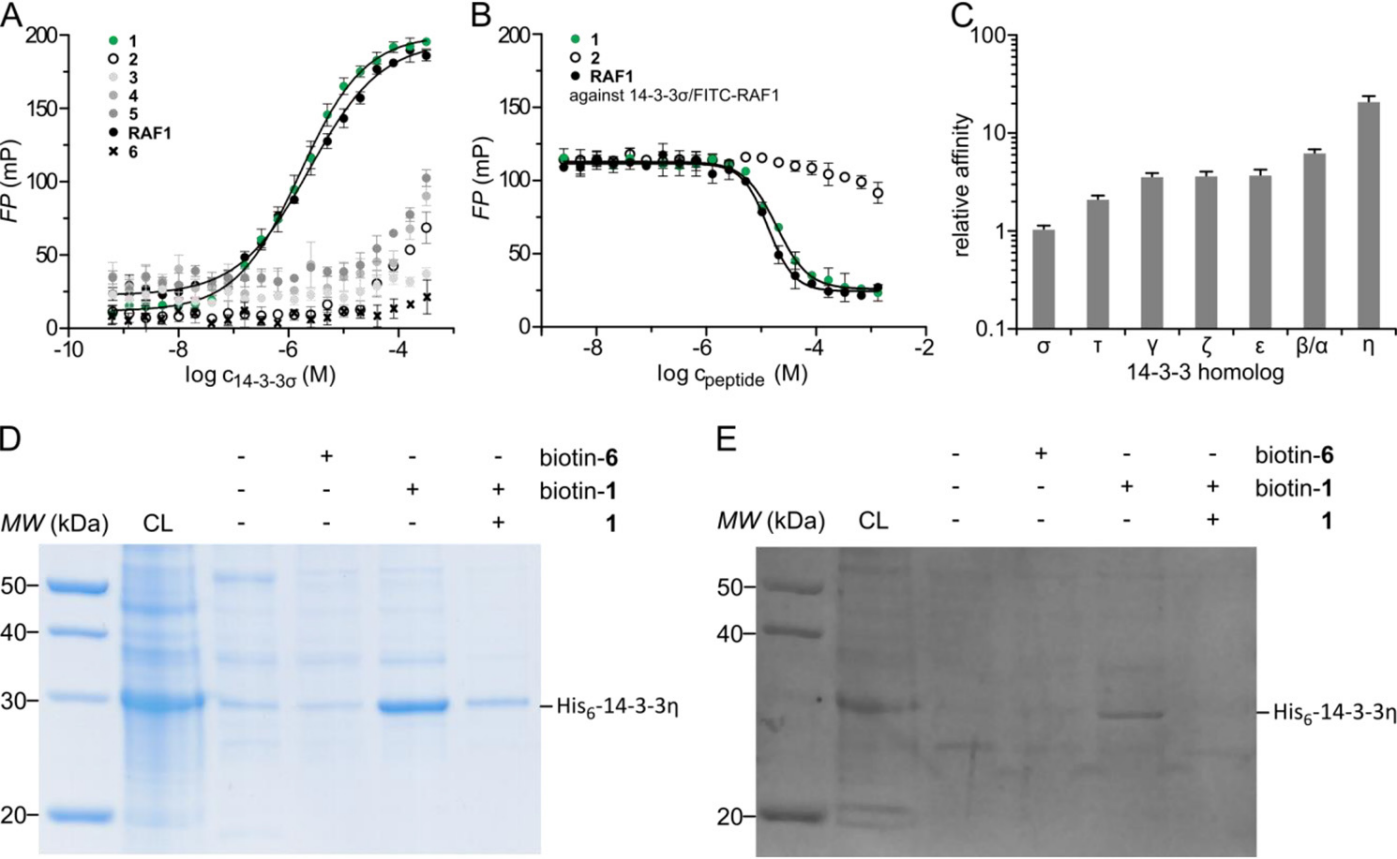
**Biochemical evaluation of 14-3-3–binding APN epitope.***A*, FP assay of 14-3-3σ titrated against FITC-labeled phospho-Ser/Thr–containing peptides (**1–6**) and the known 14-3-3–binding RAF1 epitope (mean ± S.D. (*error bars*), *n* = 3). *B*, competition of FITC-labeled RAF1 peptide from 14-3-3σ with acetylated peptides **1**, **2**, and RAF1 (mean ± S.D., *n* = 3). *C*, FP assay of different 14-3-3 homologs toward APN-binding epitope **1**. Shown are relative affinities compared with the initial tested 14-3-3σ (relative affinity = 1). *D*, Coomassie-stained SDS-PAGE of pulldown of 14-3-3η from *E. coli* cell lysate (*CL*) with biotinylated peptides **1** and **6** and competition with acetylated peptides **1**. *E*, NTA-Atto488–stained SDS-PAGE of pulldown of 14-3-3η from *E. coli* cell lysate with biotinylated peptides **1** and **6** and competition with acetylated peptide **1**.

Purified peptides were tested for direct binding toward 14-3-3σ in an FP assay, and their affinities were quantified (Fig. 2*A*). **1** was identified as the best binder (*K_D_* = 1.7 ± 0.1 μm). In addition, Ser^43^ is predicted to be potentially phosphorylated (PhosphoNET and NetPhos3.1 (41, 42)). Interestingly, this peptide contained a serine residue (Ser^44^) in the +1 position (relative to pSer^43^). To test sequence specificity and the robustness of our filtering process, the according APN 39–47 pSer^44^ peptide (**6,** KNANSpSPVA) was synthesized and analyzed in our FP assay. In contrast to **1**, **6** did not show any binding toward 14-3-3σ (Fig. 2*A*), indicating a high sequence specificity for the amino acids surrounding the phosphorylation site.

To test the influence of the used fluorophore, label-free peptides **1** and **2** were titrated against a 14-3-3σ–bound FITC-labeled RAF1 probe (FP competition assay; Fig. 2*B*). As expected from our direct FP measurements (Fig. 2*A*), **2** was not able to compete with FITC-labeled RAF1 (Fig. 2*B*). In contrast, **1** was able to compete with similar potency as the unlabeled RAF1 peptide (**1**, IC_50_ = 18.8 ± 1.5 μm; RAF1, IC_50_ = 13.4 ± 0.7 μm; Fig. 2*B*), which underlines that APN-derived motif **1** is a direct 14-3-3σ binder.

As only some homologs of 14-3-3 are currently described to be also located in the extracellular space (43), we wanted to know whether **1** is preferably bound by a subset of 14-3-3 homologs. We performed an FP assay using all human 14-3-3 homologs (β/α, γ, ε, ζ, η, and θ) and determined their relative affinities to the previously characterized 14-3-3σ. All tested homologs demonstrated an increased binding affinity, with 14-3-3β/α (5.7-fold) and 14-3-3η (18.6-fold) showing the highest preference (Fig. 2*C* and Fig. S1).

To analyze the interaction of peptide **1** with 14-3-3 proteins in an orthogonal manner, we performed a pulldown assay. To this end, we used biotinylated peptides **1** and **6** immobilized on streptavidin-agarose beads and lysates of His_6_-14-3-3β/α– or His_6_-14-3-3η–overexpressing *Escherichia coli* cells. The interacting proteins were analyzed via SDS-PAGE. All samples of the Coomassie-stained gel showed a band occurring at the expected molecular mass of His_6_-14-3-3η (∼30 kDa, Fig. 2*D*) or His_6_-14-3-3β/α (Fig. S2A) with the highest intensity for the sample containing immobilized peptide **1**. The His_6_ tag–specific NTA-Atto488 stain (Fig. 2*E* and Fig. S2B) showed that, in contrast to the negative control (6), only immobilized peptide **1** binds specifically to 14-3-3. All other bands of weaker intensities are nonspecific contaminants. Furthermore, acetylated, soluble peptide **1** was able to compete 14-3-3 proteins off the beads (Fig. 2*D* and Fig. S2), showing the specificity and the reversibility of the binding.

### Crystal structure of bound APN 38–46 pSer^43^

**Figure 3:**
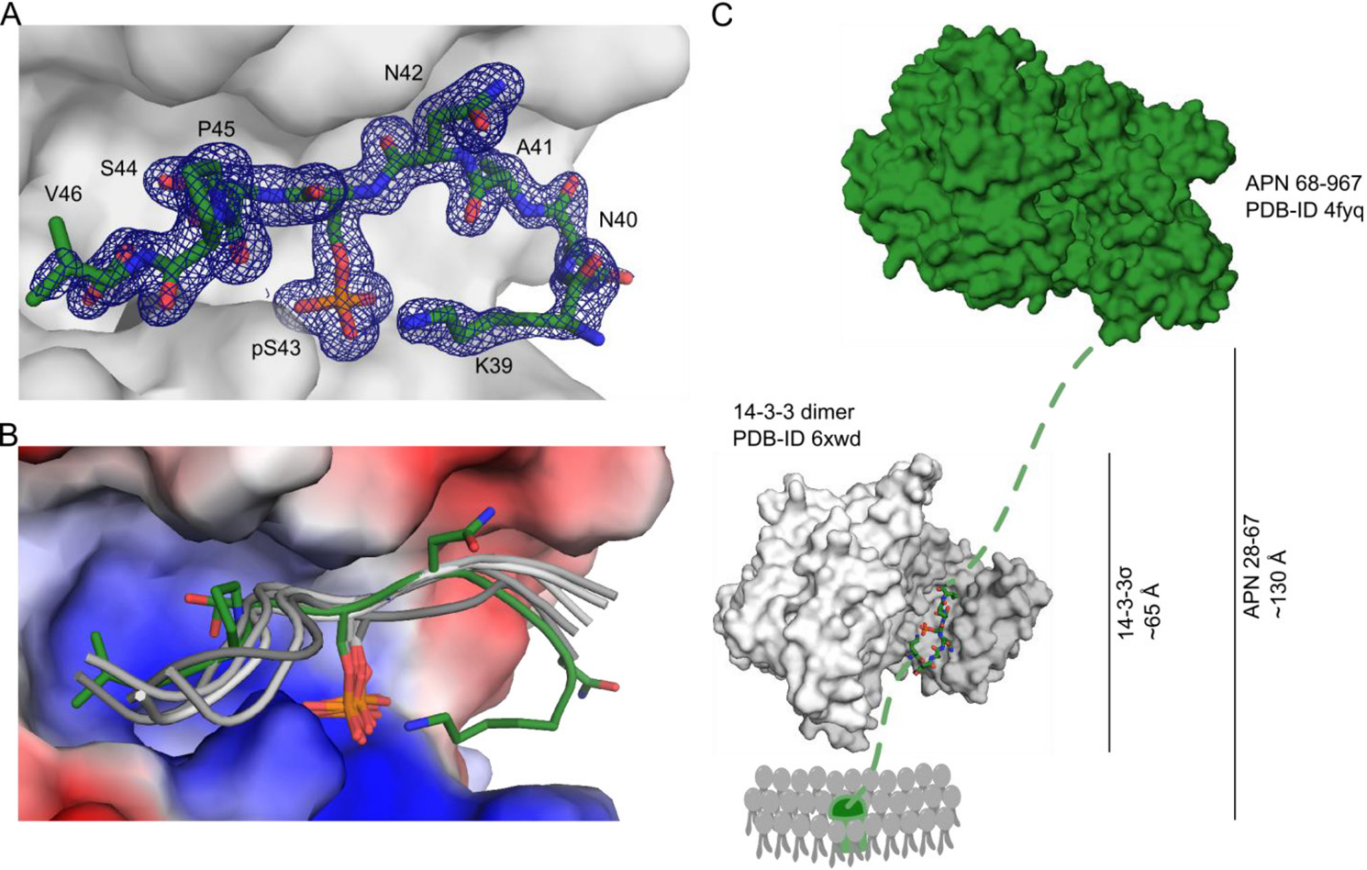
**Crystal structure of APN peptide 1 bound to 14-3-3σ.***A*, *surface representation* of our 14-3-3σ structure (PDB entry 6XWD, *light gray*) bound to APN 38–46 pSer43 (*green sticks*). The 2*F_o_* − *F_c_* composite-omit electron density map (*dark blue*, 1σ) is shown with all visible APN side chains *labeled*. *B*, overlay of 6XWD (peptide in *dark green*) with already published structures of phosphorylated peptides (*gray*) bound to 14-3-3σ (PDB entries 3MHR, 3O8I, 4FL5, 5LU2, and 5OM0 (46, 55,56, 57,58)). *C*, geometrical arrangement of crystal structures of a 14-3-3 dimer (PDB entry 6XWD, *light and dark gray surface*) in complex with peptide **1** (*green sticks*) and the extracellular domain of APN (PDB entry 4FYQ, *dark green surface* (3)). APN linker regions between the transmembrane domain and extracellular core domain are indicated (*dashed green lines*). Maximal lengths of linker regions are calculated based on the number of available amino acids.

To investigate binding of peptide **1** to 14-3-3 in more detail, we co-crystallized unlabeled peptide **1** and 14-3-3σ ΔC (aa 1–231). The collected data set was used up to a resolution of 1.60 Å (Table 1, PDB entry 6XWD), and the structure was solved in space group C222_1_ by molecular replacement (phaser (44, 45)) using a previously solved 14-3-3σ structure as search model (PDB entry 3MHR, chain A (46)). Iterative rounds of model building and refinement (Coot, Phenix (47, 48)) led to the final structural model (PDB entry 6XWD). An unbiased composite-omit electron density map clearly showed the exact location and orientation of peptide **1** within the binding groove of 14-3-3 (Fig. 3*A*). As the structure revealed one 14-3-3 monomer per asymmetric unit, the biological dimer is generated via the 2-fold crystallographic symmetry. Both binding grooves of the dimer were occupied with one peptide each.

**Table 1:** Crystal data of structure 6XWD and 7AEW Numbers in parenthesis reflect values for the highest-resolution shell.

**PDB entry**	6XWD	7AEW
**Crystal data**		
Space group	C222_1_	C222_1_
Cell dimensions		
*a*, *b*, *c* (Å)	82.0, 111.6, 62.4	82.2, 112.1, 62.6
α, β, γ (degrees)	90.0, 90.0, 90.0	90.0, 90.0, 90.0
Molecules/asymmetric unit	1	1
Wavelength (Å)	0.911650	0.911650
Resolution limits (Å)	45.37–1.60 (1.70–1.60)	45.52–1.20 (1.30–1.20)
Unique reflections	38,136 (6243)	86,519 (16,344)
Completeness (%)	100.00 (100.00)	95.8 (85.8)
Multiplicity	13.45 (13.65)	13.41 (10.16)
*I*/σ*I*	15.06 (2.66)	19.82 (2.27)
*CC*_½_	99.9 (87.9)	100.0 (85.4)
*R*_obs_ (%)	10.7 (75.4)	5.8 (81.9)
*R*_meas_ (%)	11.1 (78.4)	6.0 (85.6)
**Refinement**		
Resolution limits (Å)	45.37–1.60 (1.70–1.60)	45.52–1.20 (1.30–1.20)
*R*_work_/*R*_free_ (%)	16.76/19.62	16.18/18.36
Root mean square deviation		
Bond length (Å)	0.006	0.015
Bond angle (degrees)	0.778	1.876
*B*-factor (Å^3^)	19.97	17.76
No. of atoms		
Protein	2004	2103
Peptide	67	102
Ligand/ion	5	5
Water	419	426
Ramachandran (%)		
Favored	98.3	97.3
Allowed	1.7	2.7
Outliers	0.0	0.0

All residues for APN 39-46 could be modeled into the density. A superimposition of 14-3-3 phosphopeptide complex structures revealed that peptide **1** was bound to the common phospho-binding site with the identical N- to C-terminal orientation (Fig. 3*B*). Knowing the exact three-dimensional positioning of APN 39–46 bound to 14-3-3, we investigated the amino acid sequence up- and downstream of the phosphorylation position (pSer^43^). We were interested in whether the linker region (APN 28–67), between the plasma membrane and the globular domain of APN (aa 68–967), exposes enough space for 14-3-3 binding. Therefore, we prepared a geometrical arrangement of our 14-3-3 (PDB entry 6XWD) and the APN crystal structure (PDB entry 4FYQ (3)) and calculated the linker lengths based on the number of amino acids within the flexible linker region (Fig. 3*C*). Thereby, we could estimate the distance between the core domain and the plasma membrane to be about 130 Å. This possesses enough space for a 14-3-3 protein dimer of ∼65 Å to bind (Fig. 3*C*). The 14-3-3 dimer can either bind to a second APN molecule via the same pSer^43^ site or to the same APN molecule via an adjacent secondary APN-binding motif.

### Second noncanonical 14-3-3 motif increases affinity

**Figure 4:**
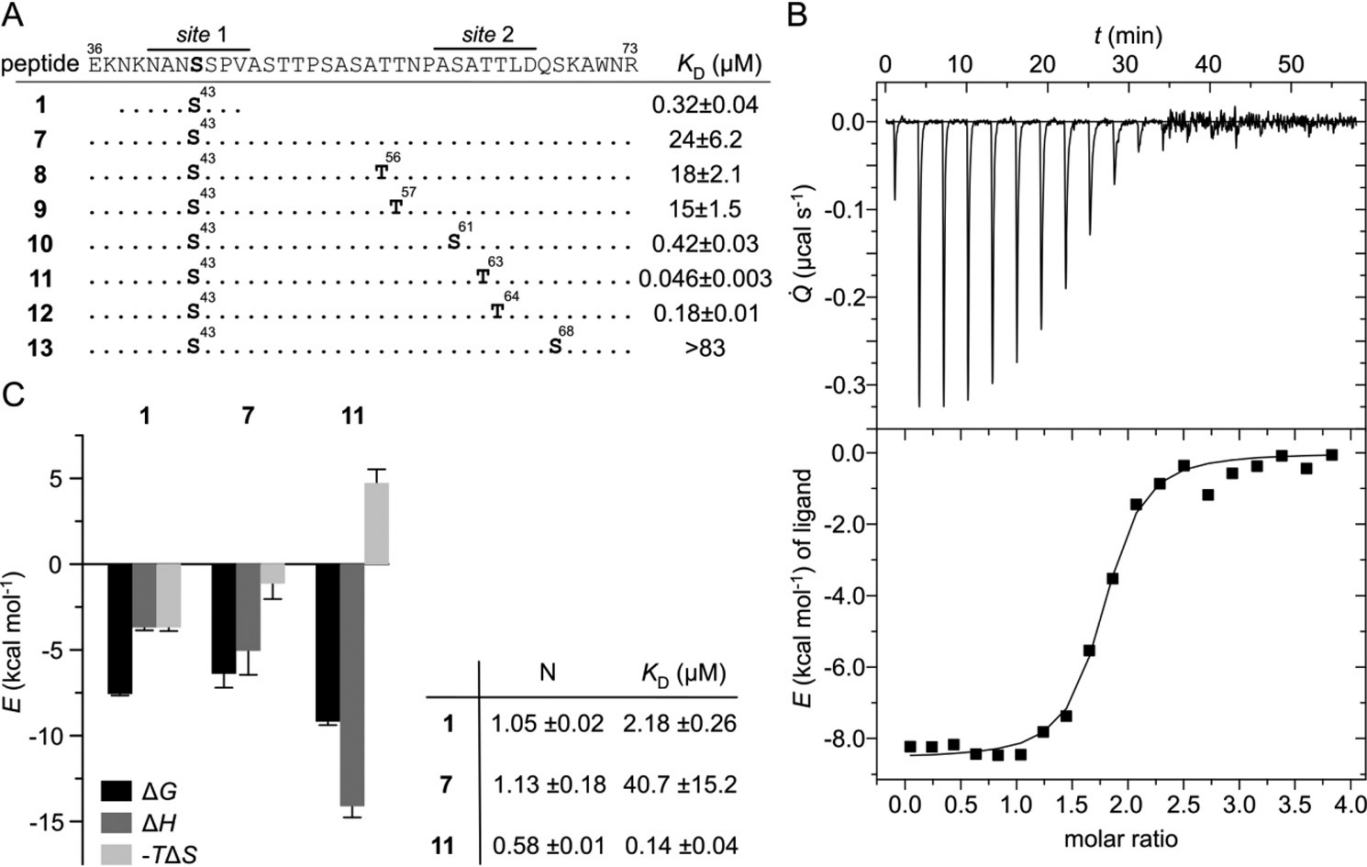
**Evaluation of noncanonical 14-3-3–binding sites.***A*, design of APN 36–73 peptides and their respective phosphorylation positions (indicated in *boldface type*). Binding affinities toward 14-3-3η (FP assay) are shown. *Black line*, identified 14-3-3–binding APN *sites 1* and *2*. *B*, ITC plot of 14-3-3η and peptide **11** (*T* = 298 K). *C*, comparison of ITC results from 14-3-3η titrated against peptides **1**, **7**, and **11**, respectively. Individual contributions of Gibbs free energy (Δ*G*), enthalpy (Δ*H*), and entropy (−*T*Δ*S*, *T* = 298 K) are shown. *Inset*, values for affinity (*K_D_*) and stoichiometry (*N*) of the binding process. Results are mean ± S.D. (*error bars*) (*n* = 3).

We investigated whether the Ser/Thr-rich residual unstructured patches of 11 N-terminal and 21 C-terminal residues of the APN linker region (Fig. 3*C*) might harbor additional noncanonical binding sites. In this region, only Thr^57^ (peptide **5**, Fig. 1) was identified as a potential canonical 14-3-3–binding site in our initial prediction analysis but did not show any relevant affinity (Fig. 2*A*). We decided to test this and all other spatial relevant potential Ser/Thr phosphorylation sites in combination with pSer43, to see whether bis-phosphorylated peptides show any change in binding affinity (Fig. 4*A*).

Fluorescently labeled peptides **7–13** (APN 36–73) were synthesized, purified, and tested for affinity toward 14-3-3β/α, -η, and -σ in FP assays. The C-terminally elongated, monophosphorylated peptide **7** showed a lower affinity (*K_D_* = 24 ± 6 μm) than the shorter, monophosphorylated peptide **1** (APN 38–46). Peptides **8** and **9** showed a comparable affinity to **7**, whereas peptides **10–12** showed an increase in binding affinity (Fig. 4*A* and Fig. S3). We observed a sequence- and distance-dependent binding when the second phosphorylation site was located within the region of Ser^61^–Thr^64^ (**10–12**) with affinities in the submicromolar range (best binder **11**: *K_D_* = 0.046 ± 0.003 μm). For these three noncanonical binding sites, the according 9-mer, monophosphorylated peptides (14, 15, 16) were synthesized, purified, and measured in our FP assay for their affinity toward 14-3-3β/α, η (Fig. S4). All three noncanonical binding sites showed affinities in the high micromolar range (*K_D_* > 40 μm) and would not be claimed to be 14-3-3 binders as such. Here, the combination of a canonical binding *site 1* (**1**, Fig. 4*A*, *K_D_* = 0.32 ± 0.04 μm) with a noncanonical binding *site 2* (**15**, Fig. S4, *K_D_* > 40 μm) resulted in an additive binding of bis-phosphorylated **11** (Fig. 4*A*, *K_D_* = 0.046 ± 0.003 μm).

We performed ITC of 14-3-3η together with **1**, **7**, **11**, and **17** (APN 36–73 pThr^63^) to investigate the thermodynamic properties of the different binding events (Fig. 4 (*B* and *C*) and Figs. S5–S8). Keeping in mind that under our experimental conditions, 14-3-3 exists as dimer in solution, we observed an increased affinity of bis-phosphorylated **11** (*K_D_* = 0.14 ± 0.04 μm) and a decreased affinity of **7** (*K_D_* = 40.7 ± 15.2 μm) compared with **1** (*K_D_* = 2.18 ± 0.26 μm, Fig. 4*C* and Figs. S5–S7), which is in accordance with our affinities determined via FP (Fig. 4*A*). For **17**, no heat change could be observed in our ITC experiment, so no affinity and thermodynamic values could be determined (Fig. S8). This is in accordance with the low affinity of peptide **15**. As expected, we observed a decreased enthalpy (Δ*H*) for the peptide **11**, due to the larger interaction surface of 38 aa *versus* 9 aa. The enthalpic contribution to binding of peptide **7** and **1** are comparable, which suggests no involvement in binding for the C-terminal region of **7**.

In addition, we observed increased −*T*Δ*S* values for **11** due to a strong entropic penalty upon binding. In the unbound state, **11** comprises higher degrees of freedom in contrast to **1**. Therefore, the relative conformational constraints upon binding show a higher influence on **11**. In contrast, with a similar length and flexibility as **11**, but only one binding epitope (*site 1*), peptide **7** shows −*T*Δ*S* values between **1** and **11**. Additionally, it is important to consider the general trend of entropy-enthalpy compensation in which the observed decrease in enthalpy causes a higher −*T*Δ*S* value.

For the binding stoichiometry, we observed similar values when comparing **1** (*n* = 1.05 ± 0.02) and **7** (*n* = 1.13 ± 0.18), which implies that one peptide binds to one 14-3-3 protomer (or two peptides bind to one 14-3-3 dimer). Most importantly, peptide **11** showed a lower stoichiometry value (*n* = 0.58 ± 0.01), indicating a bivalent binding mode in which one peptide binds to two protomers (or one 14-3-3 dimer).

To visualize the bis-phosphorylated peptide bound to 14-3-3, we co-crystallized **11** with 14-3-3σ ΔC (aa 1–231) using the vapor diffusion method in a sitting-drop setup. The collected data set was used up to a resolution of 1.20 Å (Table 1). The complex structure was solved in space group C222_1_ with one 14-3-3 monomer per asymmetric unit (PDB entry 7AEW) using molecular replacement as described earlier.

**Figure 5:**
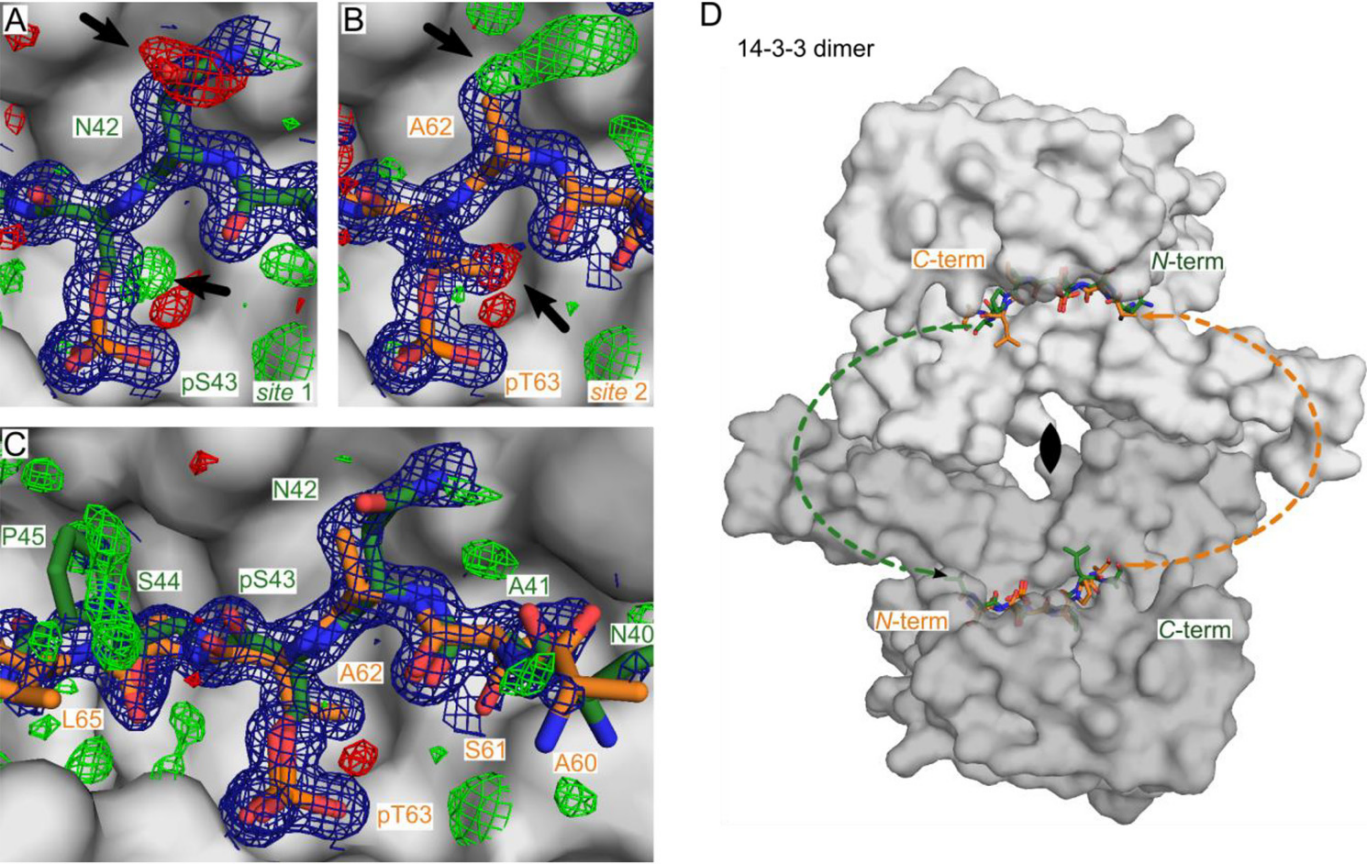
**Crystal structure of 14-3-3σ ΔC (aa 1–231)–bound bis-phosphorylated APN 36–73 pSer^43^ pThr^63^ (PDB entry**7AEW). *A*, APN *site 1* (*green sticks*) bound to 14-3-3 protomer 1 (*gray surface*). 2*F_o_* − *F_c_* electron density (*blue*, 1σ) and *F_o_* − *F_c_* differential electron density (*green* (positive), *red* (negative), 3σ) is shown around the bound APN peptide. *B*, APN *site 2* (*orange sticks*) bound to 14-3-3 protomer 1 (*gray surface*). Electron densities are shown as described in *A*. *C*, APN *site 1* and *2* (*green* and *orange sticks*) bound to 14-3-3 protomer 1. Electron densities are shown as described in *A*. *D*, 14-3-3 homodimer (*light and dark gray surface*) showing both possible orientations (*green* and *orange*) of co-crystallized APN 36–73 pSer^43^ pThr^63^. Amino acids without electron density are indicated by the *green* and *orange dashed line*. The positions of the N and C termini (*N-term* and *C-term*) are labeled.

As the biological dimer is assembled up by one of the 2-fold crystallographic symmetries, the electron density of both peptide epitopes (*site 1* and *site 2*) is averaged within the binding pocket. When building *site 1* into the binding grooves of 14-3-3, a clear positive differential density close to the C_β_-atom occurred at the pSer^43^ residue (Fig. 5*A*). Additionally, negative *F_o_* − *F_c_* density was observed around the Asn^42^ side chain (Fig. 5*A*). Similar observations were made when modeling *site 2.* Negative density was observed around the methyl group of the pThr^63^ side chain, whereas positive differential *F_o_* − *F_c_* density occurred at Ala^62^ (Fig. 5*B*).

To analyze if the bis-phosphorylated peptide **11** binds in a preferred spatial orientation within the crystal packing, we assumed no crystal symmetry and solved the same data set in space group P1. Molecular replacement resulted in two dimers (four monomers) per asymmetric unit. Careful analysis of the unbiased *F_o_* − *F_c_* differential electron density revealed similar observations as described above. This indicates that the binding grooves of the 14-3-3 dimer harbor both APN *site 1* and *site 2* simultaneously, with an overall occupancy of 50% each. The bis-phosphorylated peptide **11** binds with no preferred orientation to the 14-3-3 dimer, and solving the structure in P1 does not lead to a better structural model.

We proceeded to solve the final structure in space group C222_1_ (PDB entry 7AEW, Table 1) and could build a total of seven APN peptide residues, with ±3 amino acids relative to the phosphorylated position. C- and N-terminal residues showed only poor electron density. This could be due to peptide dynamics, especially higher flexibility of amino acids distant from the phosphorylated positions. Moreover, the exact occupancy of each peptide chain could vary from the estimated 50:50 ratio. Building a model that includes both partial occupied *sites 1* and *2* in the 14-3-3–binding cavity led to an overall better fit to the diffraction data (Fig. 5*C*). Within this model, we see that both sites bind in the commonly observed N-to-C orientation within the 14-3-3–binding pocket (Fig. 5*D*, *orange* and *green lines*). Overall, this leads to two possible orientations of the bis-phosphorylated peptide bound to the 14-3-3 dimer (Fig. 5*D* and Fig. S9).

## Discussion

The stimulation of APN by extracellular 14-3-3 is the first step in the p38 MAPK–mediated signaling pathway that regulates the induction of MMPs for tissue remodeling (11). As we are still lacking details of the initial APN-binding step, we were interested in the identification and characterization of a potential binding region for 14-3-3 in APN.

We identified five potential 14-3-3–binding sequences in the extracellular region of APN. These sequences were predicted by 14-3-3Pred (36), surface-exposed, and located in flexible, accessible looplike regions. The resulting five potential candidates (Fig. 1) were synthesized and tested in an FP assay to determine binding affinities toward 14-3-3σ, because this homolog is known be extracellular and involved in binding to APN (Fig. 2) (11). Peptide **1** (NKNANpSSPV) proved to be a strong 14-3-3 binder and was further validated by specifically competing off a fluorescently labeled RAF1 probe. This phosphorylation of APN has been predicted additionally by PhophoNET and NetPhos3.1. Because some additional 14-3-3 homologs are known to be located in the extracellular space, we tested the binding affinity of **1** against all of the other six human homologs of 14-3-3. Surprisingly, they all showed a binding affinity increase, with 14-3-3β/α and 14-3-3η showing the highest affinities (Fig. 2 and Fig. S1). These two homologs are involved in stimulation of MMPs similar to 14-3-3σ (43). A selective pulldown from cellular lysates and a thermodynamic analysis using ITC (Fig. 2, Fig. S2, and Fig. 4) further confirmed the affinity of peptide **1**. It accommodates a canonical 14-3-3 mode II binding site (*site 1*) (19).

Although the classical 14-3-3–binding modes (22) are lately discussed to be more flexible, certain positions clearly have preferences for high-affinity 14-3-3 binders, such as (but not exclusively) basic residues in positions −3 and −5, Arg/Lys in position −4, and a proline in position +2 (49). The structural analysis of **1** bound to 14-3-3σ showed the expected orientation and location of the peptide within the protein (Fig. 3, *A* and *B*) and allowed the modeling of the 14-3-3 dimer bound to the complete extracellular domain of APN (Fig. 3*C*).

This was done to elucidate whether a multivalent binding mode of 14-3-3σ to APN is possible. In addition to the high-affinity *site 1*, we could identify pThr^63^ as a second, noncanonical binding site (*site 2,* Fig. 4 and Fig. S4). When pThr^63^ was combined with *site 1,* a remarkable increase was observed in the overall affinity (∼500-fold increase of **7** *versus* **11**, Fig. 4*A* and Fig. S3). We could show that among the three epitopes increasing the overall binding affinity (10, 11, 12), **11** showed the highest-affinity gain. Similarly, from the panel of corresponding shorter 9-mer peptides (**14–16**, Fig. S4), **15** shows the highest affinity. Both **15** and **11** share the same phosphorylated Thr^63^ and the surrounding sequence stretch, which points to the direction that also noncanonical 14-3-3–binding sites show a sequence preference. The sequences of peptides **14–16** differ from canonical 14-3-3–binding sequences (*e.g.* by lacking the Arg/Lys in −4 and the Pro in the +2 position). Peptides **14** and **15** do have an Asn residue in an equal position as in peptide **1**, but the overall sequence similarity is low. It is has been described that often weaker, noncanonical sequences serve as gatekeeper sites, which enhances the overall affinity in an additive manner, often in the range of a few orders of magnitude (23, 24, 25, 26, 50, 51).

In our thermodynamic analyses (ITC), we compared peptides **7** and **11**, which have the same length and therefore a similar flexibility in the unbound state. Because **11** bears two binding sites for 14-3-3, it is more constrained upon binding than **7**, which is reflected in the higher entropic penalty. The affinity gain for peptide **11** is achieved via an enthalpic compensation due to a larger binding interface (*site 1* and *site 2*). We furthermore confirmed the binding stoichiometry of 1:1 (14-3-3 dimer/peptide **11**) (Fig. 4, *B* and *C*). Compared with a model in which two APN molecules bind to one 14-3-3 dimer via *site 1*, our data suggest a mechanism in which one 14-3-3 dimer binds to one APN molecule via *site 1* and *site 2*. When crystalizing the APN 36–73 pSer^43^ pThr^63^ bis-phosphorylated peptide with 14-3-3σ, we observed that two orientations of peptide **11** in the 14-3-3 dimer are possible. Both orientations occur within the crystal packing randomly and thereby average their occupancy (Fig. 5*D* and Fig. S9). We therefore present a protein stretch in APN capable to function as high-affinity, bivalent binder to 14-3-3.

Taken together, we identified Ser^43^ as a canonical, high-affinity binding *site 1* and an additional, noncanonical gatekeeper binding *site 2*, which resulted in a remarkable affinity gain of about 500-fold. Crystal structure analysis resulted in two 14-3-3 structures comparing a monophosphorylated (PDB entry 6XWD) and a bis-phosphorylated APN stretch (PDB entry 7AEW). Our work adds to the upcoming field of bivalent 14-3-3 binders and emphasizes especially the importance of in-depth analysis of 14-3-3 secondary gatekeeper sites. Furthermore, we could shed some light on the first steps of 14-3-3–driven APN signal transduction. Further research is needed to show the cellular effect of these phosphorylation sites in APN.

## Experimental procedures

### Prediction of 14-3-3–binding sites in APN

Prediction of possible phosphorylated binding epitopes was initially done by 14-3-3Pred using the sequence of the human aminopeptidase N (UniProt: P15144 (36)). The according potential binding motifs above threshold (artificial neural network ≥ 0.55, position-specific scoring matrix ≥ 0.8, support vector machines ≥ 0.25) were further analyzed for their surface accessibility (PDB entry 4FYQ (3)) using Swiss PDB Viewer (37). Additionally, the localization within secondary structure elements was analyzed using DSSP (38, 39, 40). Only amino acids that (i) fulfilled at least one 14-3-3Pred threshold and (ii) showed a minimal surface accessibility of 20% and (iii) were not part in a α-helix or a β-sheet were taken into further consideration.

### Peptide synthesis and purification

All peptides were synthesized using standard Fmoc-based SPPS on an automated peptide synthesizer (Biotage Syro I). Initial loading of the 2-chlorotrityl resin was performed manually in dichloromethane using 1 eq of amino acid with 10 eq of *N*,*N*-diisopropylethylamine (DIPEA) for 60 min. Residual unreacted resin was capped using methanol/DIPEA/dichloromethane (2:1:7) for 10 min. Automated SPPS consisted of four repetitive steps. Removal of the Fmoc protecting group was performed using 25% piperidine in dimethylformamide (DMF) twice for 5 min. Coupling of amino acids was done by first using 4 eq of amino acid, 4 eq of benzotriazol-1-yl-oxytripyrrolidinophosphonium hexafluorophosphate, 4 eq of Oxyma, and 8 eq of DIPEA in DMF/*N*-methylpyrrolidone for 25 min and in a second reaction 4 eq of amino acid, 4 eq of 1-[bis(dimethylamino)methylene]-1H-1,2,3-triazolo[4,5-b]pyridinium 3-oxide hexafluorophosphate, 4 eq of Oxyma, and 8 eq of DIPEA in DMF/*N*-methylpyrrolidone for 25 min. Acetylation of unreacted amines was performed using 10% acetic anhydride in DMF for 5 min. For fluorescent labeling (PEG2) and biotinylation (PEG4), the peptides were first equipped with a PEG linker under amino acid–coupling conditions. FITC or biotin was added by using 4 eq of the reactant and 8 eq of DIPEA in DMF twice for 60 min. For N-terminally acetylated peptides, a solution of acetic anhydride/DIPEA/DMF (8:1:1) was added to the peptide after removal of the Fmoc protecting group. Final cleavage from resin was performed with a solution of 94% TFA, 2.5% H_2_O, 2.5% 1-octadecanethiol, and 1% triisopropylsilane for 3 times 1 h. After evaporation of the TFA by N_2_ stream, peptides were precipitated with diethylether at −20 °C, redissolved in 1:1 acetonitrile/water, and lyophilized.

Purification of peptides was performed using reversed-phase semipreparative HPLC (Agilent) with a Nucleodur C18 reverse-phase column (125 × 10 mm, particle size 5 μm; Macherey-Nagel) using solvents A (water + 0.1% TFA) and B (acetonitrile + 0.1% TFA) with a flow rate of 6 ml min^−1^. Characterization of peptides was performed using an HPLC/electrospray ionization-MS (Agilent) system with a reversed-phase C18 column (ZORBAX Eclipse XDB-C18, 4.6 × 150 mm, particle size 5 μm) and the above-mentioned solvents A and B (Figs. S10–S14).

Quantification of FITC-labeled peptides was done by measuring absorbance using the NanoDrop system in a 100 mm Na_3_PO_4_ buffer at pH 8.5 (λ = 495 nm, ε = 77,000 m^−1^ cm^−1^). Peptides containing a tryptophan residue were quantified by measuring absorbance using the NanoDrop system (λ = 280 nm, ε = 5690 m^−1^ cm^−1^). All other peptides were quantified using HPLC quantification via peak integration and a reference peptide (λ = 210 nm).

### Protein expression

N-terminally His_6_-tagged 14-3-3 full-length proteins were expressed with heterologous bacterial expression. *E. coli* BL21(*DE3*) was chemically transformed with pProEx_HTb vector containing the protein-coding gene and grown in TB medium at 37 °C until *A*_600_ = 1.3–1.5 was reached. Protein expression was induced using 0.5 mm isopropylthiogalactoside, the culture was incubated overnight at 25 °C, 150 rpm. Cells were harvested at 4000 rcf, 4 °C, 20 min, and the cell pellet was dissolved in lysis buffer (50 mm Tris, pH 8.0, 300 mm NaCl, 5% glycerol, 10 mm imidazole, 1 mm β-mercaptoethanol). Unless otherwise stated, the next steps were performed at 4 °C. For cell disruption, 100 μm phenylmethylsulfonyl fluoride, lysozyme, and DNase I were added, and cells were disrupted using a Microfluidizer (Microfluidics Microfluidizer LM10, H10Z 100-μm shearing cell). The lysate was cleared via ultracentrifugation 53,350 rcf, 45 min), and the supernatant was loaded on a 5-ml HisTrap FF crude column (GE Life Sciences) pre-equilibrated with lysis buffer. The column was washed with 8 column volumes of wash buffer (50 mm Tris, pH 8.0, 500 mm NaCl, 5% glycerol, 25 mm imidazole, 1 mm β-mercaptoethanol), and protein elution was performed with 4 column volumes of elution buffer (50 mm Tris, pH 8.0, 200 mm NaCl, 5% glycerol, 250 mm imidazole, 1 mm β-mercaptoethanol). The protein solution was concentrated using a 10-kDa cut-off ultrafiltration device (Amicon, Merck Millipore), and final purification was performed using size exclusion chromatography (SEC, HiLoad 16/600 Superdex 75 pg, GE Healthcare) on an ÄKTA Pure FPLC system with SEC buffer (10 mm HEPES, pH 8.0, 150 mm NaCl, 2 mm DTT). After concentration via ultrafiltration as described above up to ∼50 g liter^−1^, the pure protein was flash-frozen in liquid nitrogen and stored at −80 °C.

C-terminally truncated protein (14-3-3 ΔC aa 1–231) was expressed and purified as mentioned above. After elution of the protein from the HisTrap FF crude column, protein was dialyzed using dialysis buffer (50 mm Tris, pH 8.0, 100 mm NaCl, 1 mm β-mercaptoethanol) and simultaneously proteolytically cleaved using tobacco etch virus protease in an overnight reaction. The protein solution was circulated over a 5-ml HisTrap FF crude column for 1 h. Final purification was performed using SEC (HiLoad 16/600 Superdex 75 pg on an ÄKTA Pure FPLC system, both from GE Healthcare) with SEC buffer. After concentration via ultrafiltration as described above, up to 30 g liter^−1^, the pure protein was flash-frozen in liquid nitrogen and stored at −80 °C.

### FP assay

For the FP assay, the 14-3-3 proteins were diluted to a protein-specific start concentration in FP buffer (10 mm HEPES, pH 7.4, 150 mm NaCl, 2 mm DTT, 0.01% Tween 20). DMSO stocks of peptides were diluted in FP buffer to a concentration of 30 nm. For the assay, 10 μl of FP buffer were pipetted in 20 wells of a 384-well Corning black round bottom plate. A dilution series with 10 μl of protein solution was performed, and 5 μl of peptide solution was added to each well for a final volume of 15 μl. The plate was incubated for 60 min at room temperature. Measurement was performed at 25 °C in a Tecan Spark20M plate reader (λ_ex_ = 470 nm, λ_em_ = 525 nm). Data analysis was performed with GraphPad Prism (version 5.03), and *K_D_* values were determined via a nonlinear regression fit of dose-response curves with variable slope (four parameters).

### Fluorescence polarization competition assay

The fluorescence polarization competition assay was prepared by diluting the 14-3-3σ protein to 3 μm with FP buffer (10 mm HEPES, pH 7.4, 150 mm NaCl, 2 mm DTT, 0.01% Tween 20) and adding 30 nm FITC-labeled RAF1 peptide (tracer). 10 μl of FP buffer was pipetted in 20 wells of a 384-well Corning black round bottom plate, and a dilution series with 10 μl of acetylated peptide **1** (starting concentration 4000 μm) was performed. 5 μl of the premixed complex of 14-3-3 protein and tracer peptide was added to the dilution series to a total volume of 15 μl. The plate was incubated at room temperature for 60 min, and the measurement was performed at 25 °C using a Tecan Spark20M plate reader (λ_ex_ = 470 nm, λ_em_ = 525 nm). Data analysis was performed with GraphPad Prism (version 5.03), and *K_D_* values were determined via a nonlinear regression fit of dose-response curves with variable slope (four parameters).

### Pulldown assay

14-3-3–overexpressing *E. coli* cell lysate was prepared by lysing *E. coli* cells via sonication in pulldown buffer and removing cell debris by ultracentrifugation (53,350 rcf, 45 min, 4 °C). 24 nmol of Streptavidin Sepharose High Performance beads (GE Healthcare) were equilibrated with pulldown buffer (50 mm HEPES, pH 7.4, 250 mm NaCl, 20 mm MgCl_2_) and incubated with 24 nmol of biotin-**1**, biotin-**6**, or no peptide for 45 min on ice. Afterward, the beads were washed three times with pulldown buffer. Subsequently, the beads were incubated for 120 min in the prepared cell lysate. After incubation, beads were washed three times with pulldown buffer and split in half either for direct SDS-PAGE or for competition analysis, respectively. For competition, beads incubated with biotin-**1** were incubated with 96 nmol of acetylated peptide **1** for 60 min and washed three times with pulldown buffer. For SDS-PAGE, 6 nmol of beads were added per lane on a Tris-Tricine (52) gel and run for 120 min at 150 V. After electrophoresis, gel was fixated for 90 min in a solution of 50% water, 40% EtOH, and 10% acetic acid and consequently washed two times for 30 min in water. Staining of the His_6_-tagged proteins in the gel was performed in a 1:1000 dilution of a 1 μg/μl solution of NTA-Atto488 (Sigma–Aldrich) for 60 min. Access stain was removed by two washing steps, each 60 min, in water. Images were taken with a Bio-Rad Gel Doc XR+ Gel documentation system. Coomassie staining was performed afterward on the same gels.

### Protein crystallization and X-ray structure determination

Crystals of 14-3-3σ ΔC (aa 1–231) with peptide **1** were obtained by mixing 12.5 g liter^−1^ protein in a molar 1:2 ratio with peptide. 100 nl of the co-crystallization solution was mixed with 100 nl of crystallization solution (100 mm HEPES, pH 7.3, 0.19 m CaCl_2_, 5% glycerol, 28% PEG400) and incubated as sitting drops in iQ-3-well plates (TTP) at 4 °C. Crystals of 14-3-3σ ΔC (aa 1–231) and peptide **11** were obtained in the same manner as mentioned above, but 100 mm HEPES, pH 7.7, 0.19 m CaCl_2_, 5% glycerol, 27% PEG400 was used for crystallization. Crystals appeared after 2–3 days, were fished into nylon loops (Hampton), and were immediately flash-frozen in liquid nitrogen.

X-ray diffraction measurement was performed at the I04 beamline at the Diamond Light Source (DLS, Didcot, Oxfordshire, UK). Integration of data sets was performed using XDS (53). Molecular replacement was performed with Phaser MR from the CCP4i software suite (44) by using PDB entry 3MHR (46) as the search model. Model building and refinement was done using Coot (47) and Phenix (48) or Refmac5 (54) for refinement.

### ITC

The ITC experiments were performed on an automated MicroCal iTC200 (Malvern Panalytical). To prepare 14-3-3 proteins for ITC measurements, a buffer exchange to ITC buffer (50 mm HEPES, pH 7.4, 250 mm NaCl, 20 mm MgCl_2_) was performed on a Superdex 75 10/300 GL column (ÄKTA Pure FPLC system, GE Healthcare). For peptides **1**, **7**, and **17**, a 500 μm solution of protein was titrated to a 50 μm solution of peptide in 18 steps (2-μl volume in 4 s, 180-s spacing) at 25 °C. For peptide **11**, a 250 μm solution of protein was titrated to a 12.5 μm solution of peptide with the settings mentioned before. The reference power was set to 6 kcal s^−1^, and the stirring speed was set to 750 rpm. Analysis was performed using MicroCal ITC-ORIGIN analysis software.

## Data availability

All structures in this paper have been deposited in the PDB and can be found with the following codes: 6XWD and 7AEW. All remaining data are contained within the article and the supporting information.
