# Supplementary material for: MMP activation–associated aminopeptidase N reveals a bivalent 14-3-3 binding motif
Source: J Biol Chem. 2021 Jan 13;295(52):18266–75. doi: 10.1074/jbc.RA120.014708 (PMC7939381; doi:10.1074/jbc.RA120.014708)
Supplement: Supplementary file 1 [file mmc1.pdf]

Supplementary Information

MMP activation associated Aminopeptidase N reveals a bivalent 14-3-3 binding motif

Sebastian Kiehstaller[1,2], Christian Ottmann[3], Sven Hennig[1,2]*

[1] Division of Organic Chemistry, Department of Chemistry and Pharmaceutical Sciences, Vrije Universiteit Amsterdam, 1081 HZ Amsterdam, Noord-Holland, Netherlands.

[2] Amsterdam Institute of Molecular and Life Sciences (AIMMS), VU University Amsterdam, Amsterdam, Netherlands

[3] Laboratory of Chemical Biology, Department of Biomedical Engineering and Institute for Complex Molecular Systems, Eindhoven University of Technology, Eindhoven, Noord-Brabant, Netherlands.

*Corresponding author: Sven Hennig

Figure S1. Fluorescence polarization assay of all human 14-3-3 homologs against peptide **1**.

Figure S2. Pull-down of 14-3-3.

Figure S3. Design and FP titrations of 14-3-3 against bis-phosphorylated APN 36-73 peptides **7-13**.

Figure S4. Fluorescence polarization assay of 7-mer peptides **14-16** containing phosphorylation sites pSer61, pThr63 and pThr64 respectively.

Figure S5. Triplicate ITC measurements of 14-3-3 and peptides **1**.

Figure S6. Triplicate ITC measurements of 14-3-3 and peptides **11**.

Figure S7. Triplicate ITC measurements of 14-3-3 and peptides **7**.

Figure S8. Triplicate ITC measurements of 14-3-3 and peptides **17**.

Figure S9. Possible APN 36-73 pSer43 pThr63 orientations.

Figure S10. HPLC/ESI-MS analysis.

Figure S11. HPLC/ESI-MS analysis.

Figure S12. HPLC/ESI-MS analysis.

Figure S13. HPLC/ESI-MS analysis.

Figure S14. HPLC/ESI-MS analysis.

Table S1. 14-3-3Pred results.

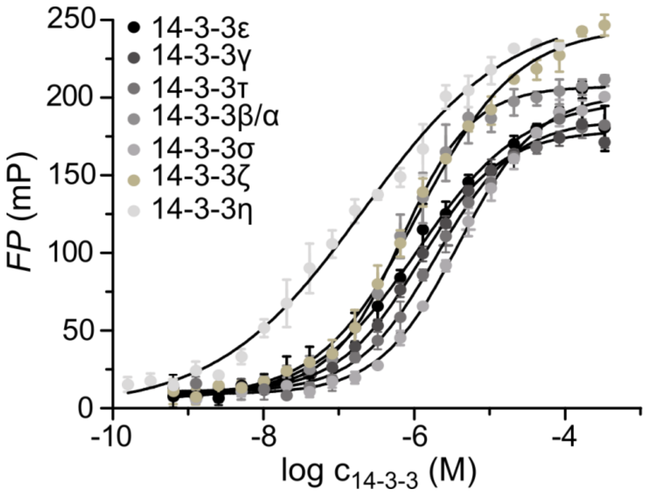

homolog	K_D (µM)
14-3-3ε	1.1±0.1
14-3-3γ	1.16±0.07
14-3-3τ	2.0±0.1
14-3-3β/α	0.7±0.0.5
14-3-3σ	3.9±0.2
14-3-3ζ	1.2±0.1
14-3-3η	0.21±0.03

Figure S1. Fluorescence polarization assay of all human 14-3-3 homologs against peptide **1**. Table shows resulting binding affinities (K_D). (Mean+SD, n=3).

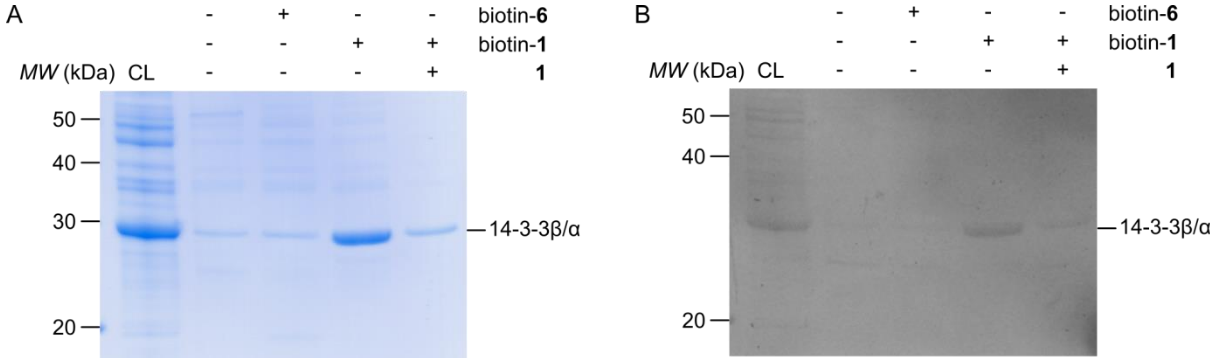

Figure S2. Pull-down of 14-3-3**.** Pull down of 14-3-3β/α from *E. coli* cell lysate with biotinylated peptides **1**, **6** and competition with acetylated peptides **1**. (A) Coomassie stained SDS-PAGE. (B) NTA-Atto488 stained SDS-PAGE.

A

peptide	^{36}EKNKNANSSPVASTTPSASATTNPASATTLDQSKAWNR^{73}	K_D (µM) 14-3-3η	14-3-3β/α	14-3-3σ
**1**	.....**S**^{43}...	0.32 ± 0.04	0.71 ± 0.04	3.5 ± 0.22
**7**	.......**S**^{43}..............................	24 ± 6.2	60 ± 20	>167
**8**	.......**S**^{43}............**T**^{56}.................	18 ± 2.1	32 ± 5.5	>167
**9**	.......**S**^{43}.............**T**^{57}................	15 ± 1.5	23 ± 4.7	>167
**10**	.......**S**^{43}.................**S**^{61}............	0.42 ± 0.03	0.88 ± 0.09	6.6 ± 1.4
**11**	.......**S**^{43}...................**T**^{63}..........	0.046 ± 0.003	0.11 ± 0.01	1.8 ± 0.13
**12**	.......**S**^{43}....................**T**^{64}.........	0.18 ± 0.01	0.45 ± 0.04	4.1 ± 0.31
**13**	.......**S**^{43}........................**S**^{68}.....	>83	>167	>167

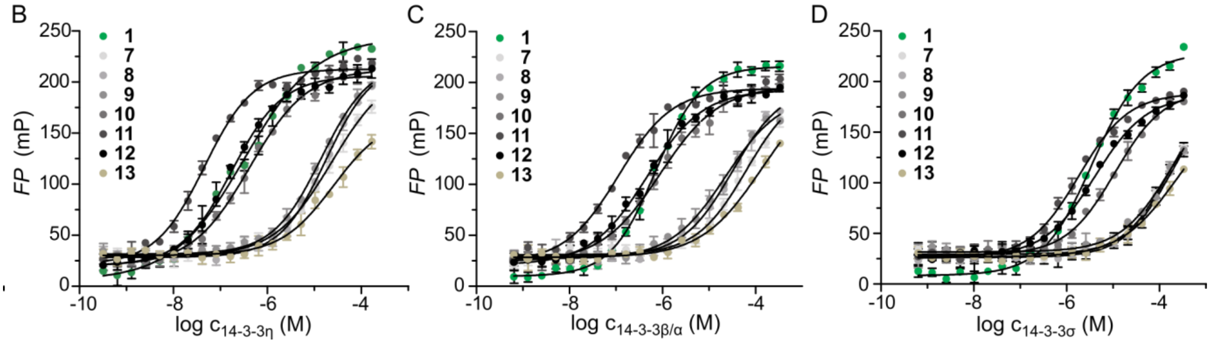

Figure S3. Design and FP titrations of 14-3-3 against bis-phosphorylated APN 36-73 peptides **7-13.** (A) Design of bis-phosphorylated APN peptides. All peptides were FITC labeled for FP assays. Bold letters indicate phosphorylation position. Binding affinities (K_Ds in µM) are shown for 14-3-3 homologs (β/α, η, σ). (B) FP titrations of 14-3-3η against bis-phosphorylated APN 36-73 peptides **7-13**. Peptide **1** measured for comparison. (C) Same as B with 14-3-3β/α. (D) Same as B with 14-3-3σ. (Mean+SD, n=3).

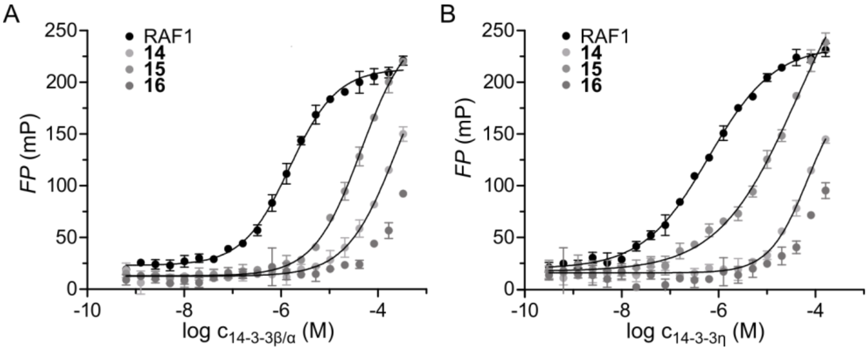

C

K_D (µM)	14	15	16
14-3-3β/α	>167	45±4.5	>333
14-3-3η	70±20	40±20	>167

Figure S4. Fluorescence polarization assay of 9-mer peptides **14-16** containing phosphorylation sites pSer61, pThr63 and pThr64 respectively. (A) FP titration of 14-3-3β/α against peptides **14-16**. Technical positive control: **RAF1** peptide. (B) Same as A, with 14-3-3η. (C) Estimated binding affinities of **A** and **B**. (Mean+SD, n=3).

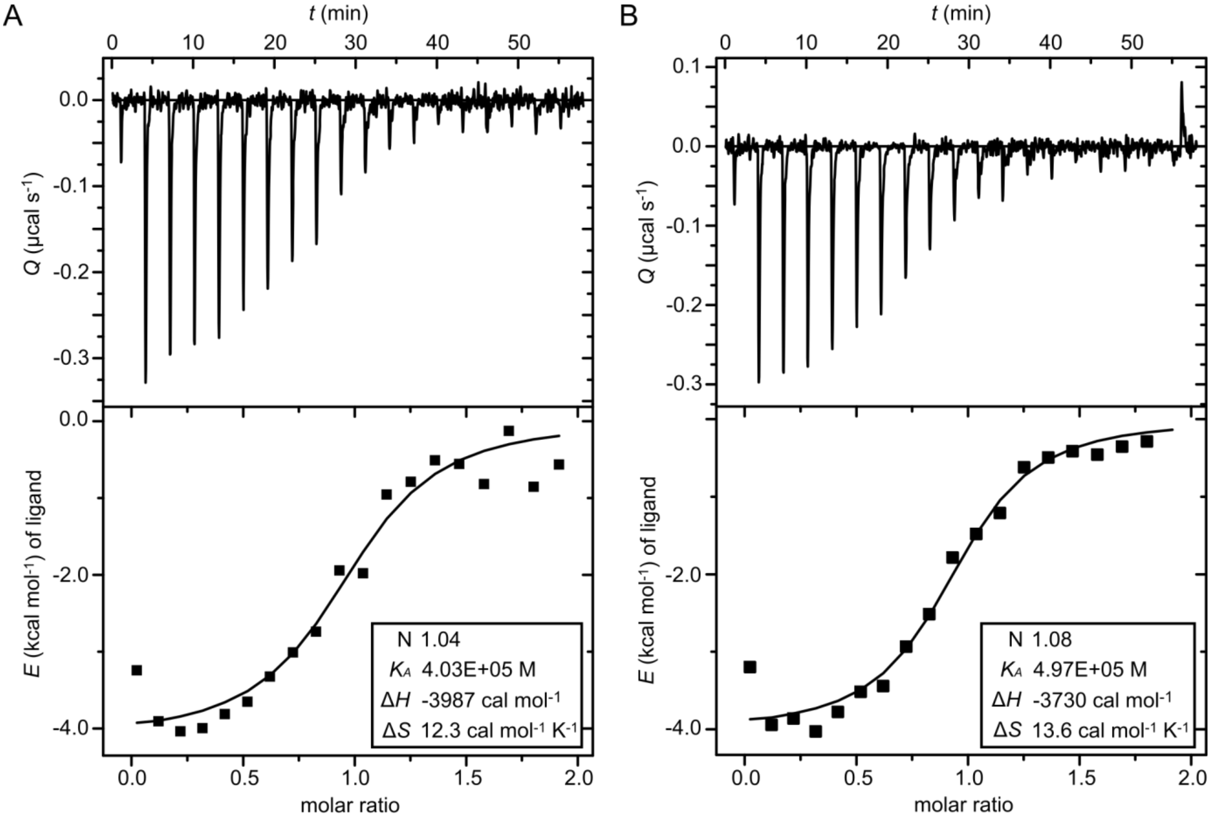

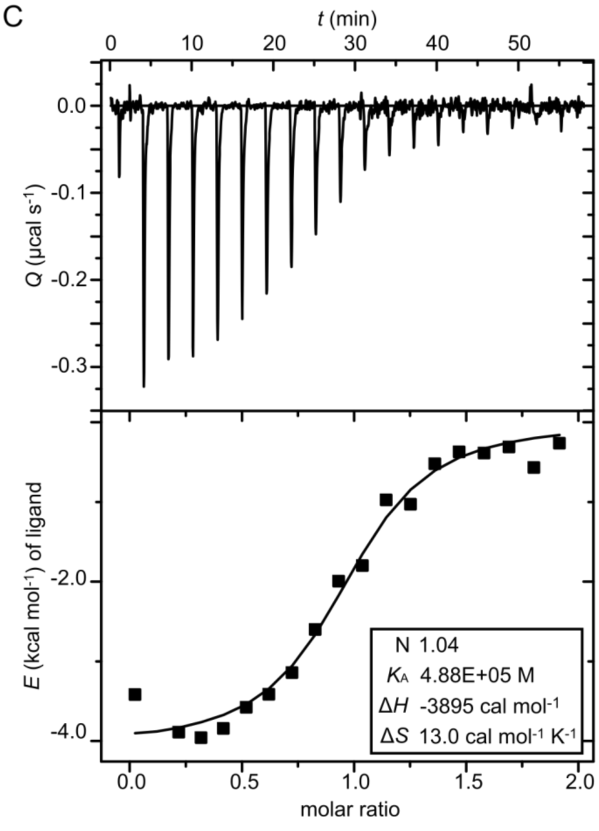

D

	K_D (µM)	ΔG (cal mol^{-1})
A	2.48	-7652.4
B	2.01	-7782.8
C	2.05	-7769.0

Figure S5. Triplicate ITC measurements of 14-3-3 and peptides **1.** (A-C) Triplicate ITC measurements of peptide **1** including values for stoichiometry (N), association constant (K_A), changes in Enthalpy (ΔH), changes in Entropy (ΔS). (D) Calculated values for dissociation constant (K_D) and changes in Gibbs free energy (ΔG).

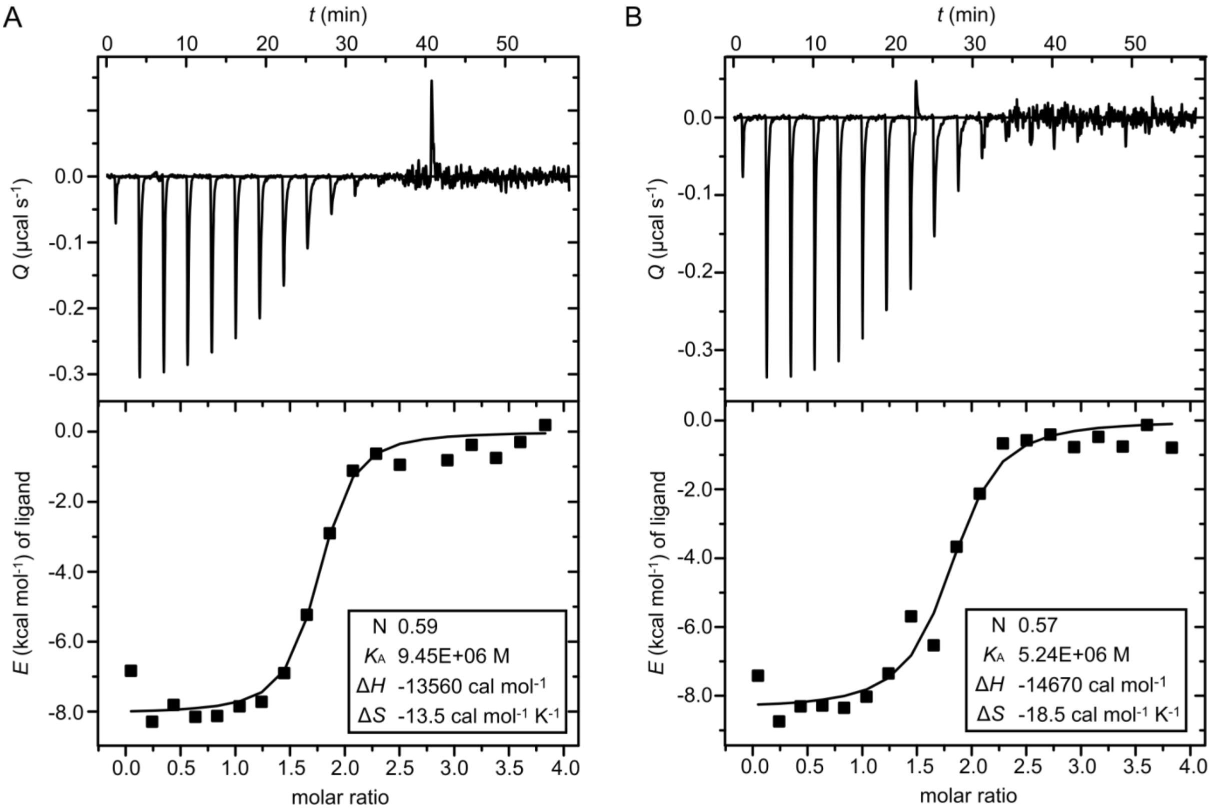

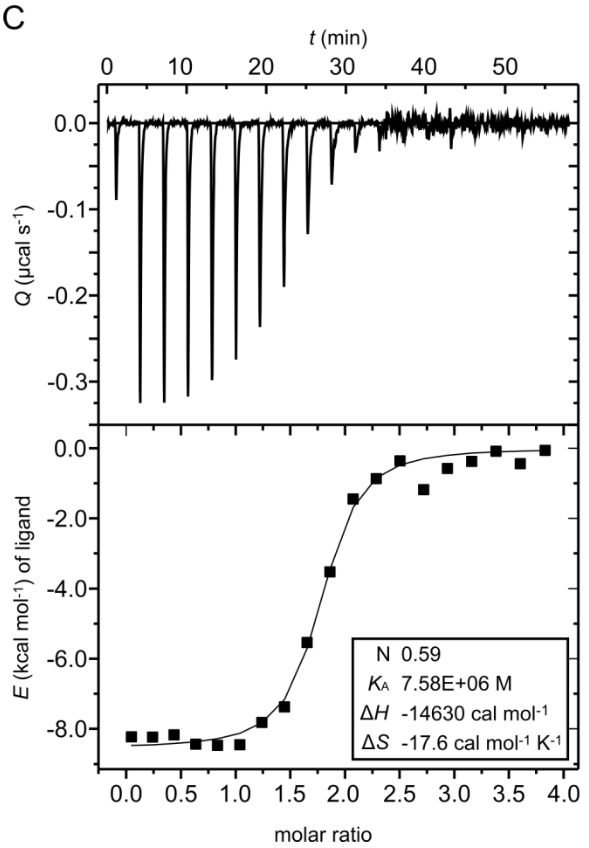

D

	K_D (µM)	ΔG (cal mol^{-1})
A	0.11	-9507.2
B	0.19	-9157.0
C	0.13	-9385.2

Figure S6. Triplicate ITC measurements of 14-3-3 and peptides **11.** (A-C) Triplicate ITC measurements of peptide **11** including values for stoichiometry (N), association constant (K_A), changes in Enthalpy (ΔH), changes in Entropy (ΔS). (D) Calculated values for dissociation constant (K_D) and changes in Gibbs free energy (ΔG).

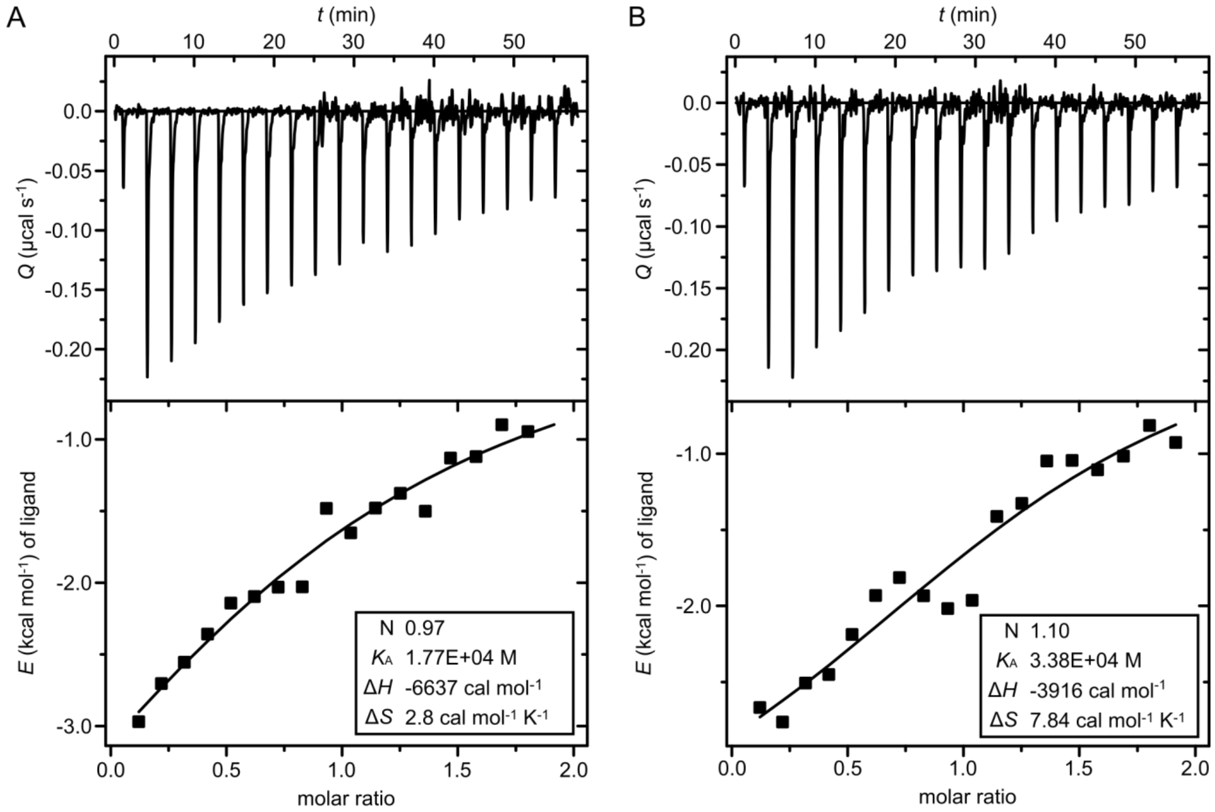

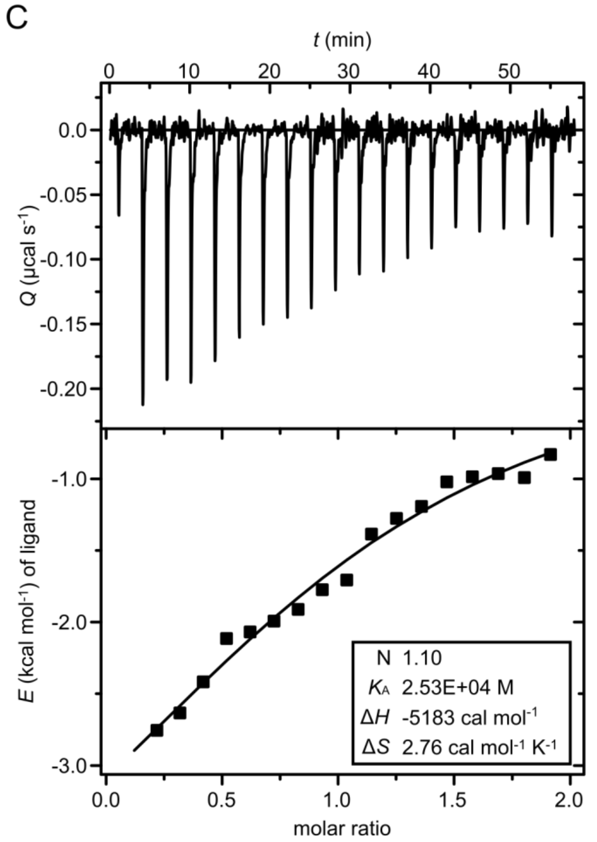

D

	K_D (µM)	ΔG (cal mol^{-1})
A	56.5	-7477.4
B	39.5	-6252.3
C	26.1	-6005.5

Figure S7. Triplicate ITC measurements of 14-3-3 and peptides **7.** (A-C) Triplicate ITC measurements of peptide **7** including values for stoichiometry (N), association constant (K_A), changes in Enthalpy (ΔH), changes in Entropy (ΔS). (D) Calculated values for dissociation constant (K_D) and changes in Gibbs free energy (ΔG).

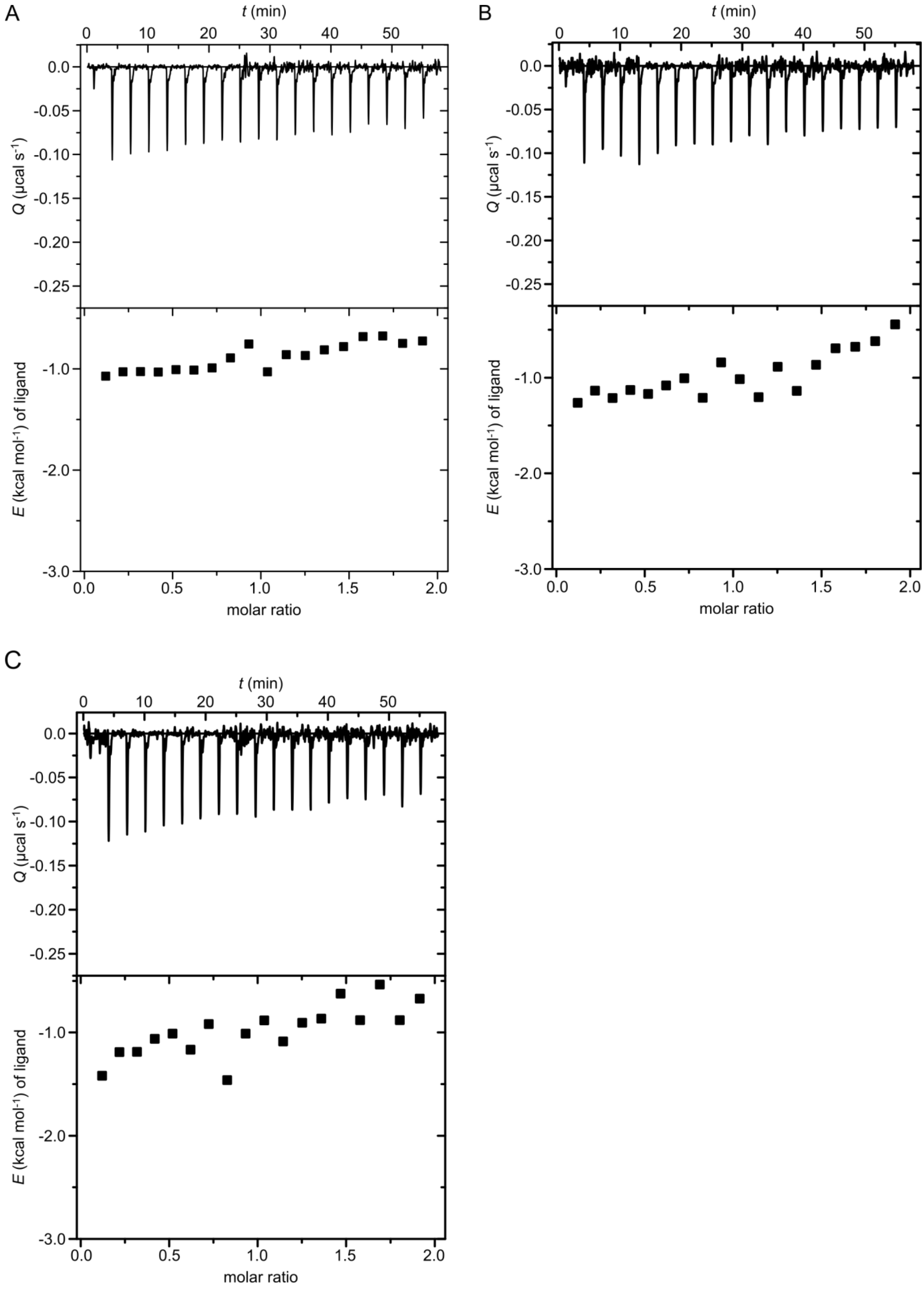

Figure S8. Triplicate ITC measurements of 14-3-3 and peptides **17.** (A-C) Triplicate ITC measurements of peptide **17**.

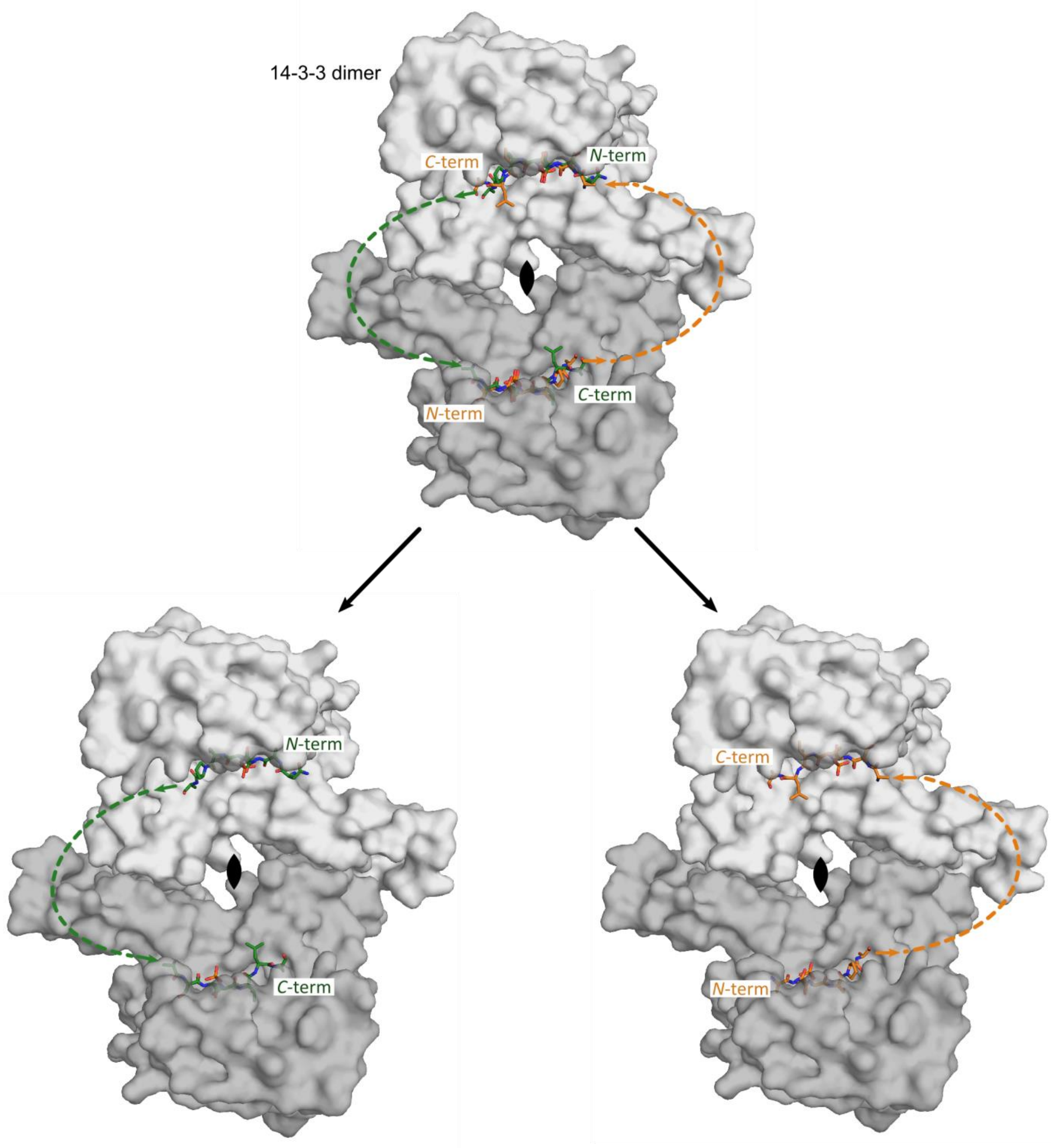

Figure S9. Possible APN 36-73 pSer43 pThr63 orientations**.** Crystal structure of the 14-3-3σ homodimer (light- and dark-grey surface). Both orientations of the APN peptide (orange and green sticks) build in the 14-3-3 homodimer (light- and dark-grey surface). Both individual peptide orientations are separated in the lower row for a better overview. Structurally unresolved connection linker is indicated as dashed line. APN *N*- and *C*-terminus is labeled. The two-fold crystallographic symmetry is indicated (black oval).

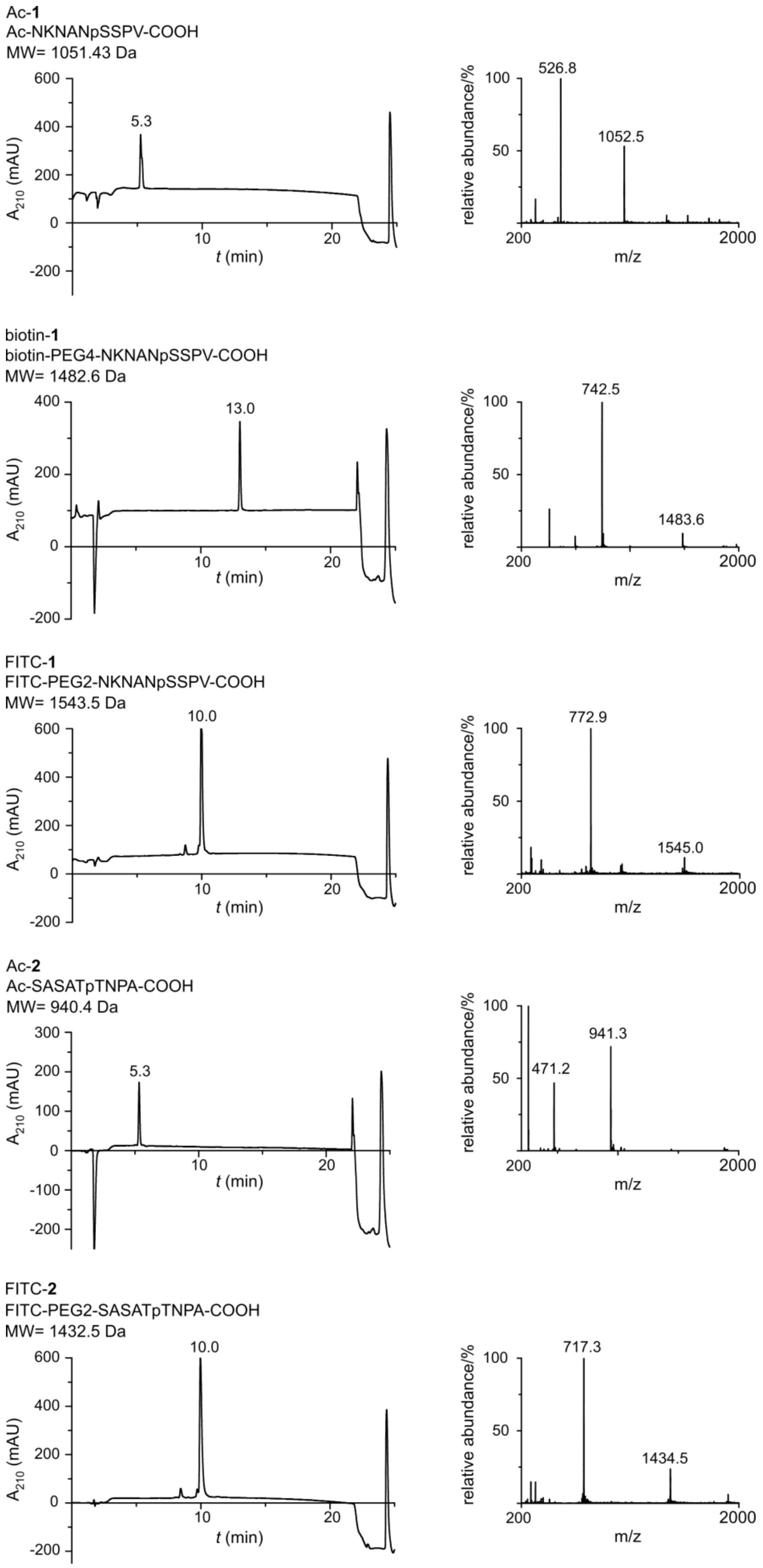

Figure S10. HPLC/ESI-MS analysis. Analysis of peptides Ac-**1**, biotin-**1**, FITC-**1**, Ac-**2** and FITC-**2** performed on Agilent HPLC system using ZORBAX Eclipse XDB-C18 column. Left chromatogram shows Absorption (A_{210}) over time (t) and right diagram show relative abundance of MS-ions.

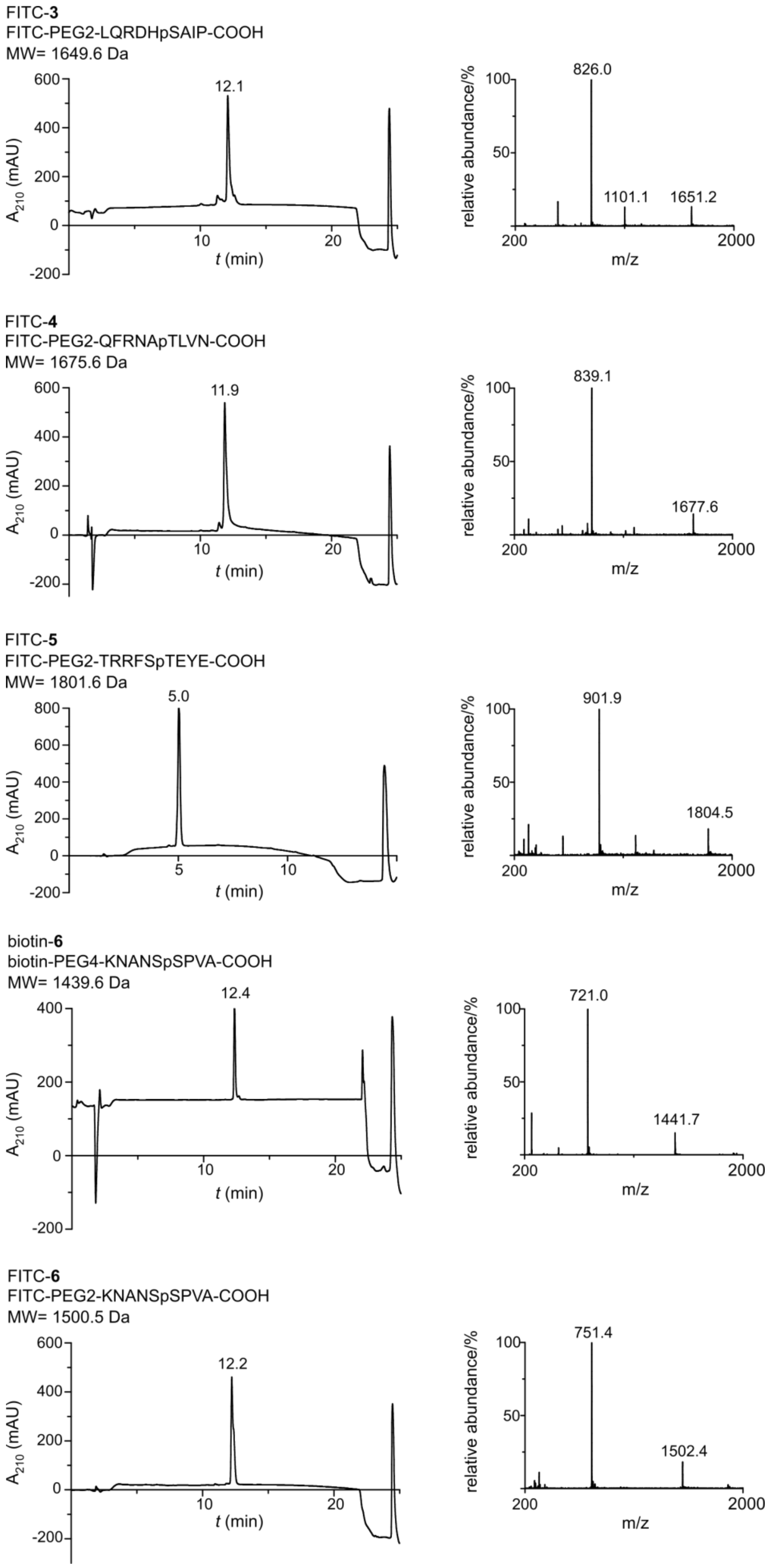

Figure S11. HPLC/ESI-MS analysis. Analysis of peptides FITC-**3**, FITC-**4**, FITC-**5**, biotin-**6** and FITC-**6** performed on Agilent HPLC system using ZORBAX Eclipse XDB-C18 column. Left chromatogram shows Absorption (A_{210}) over time (t) and right diagram show relative abundance of MS-ions.

Ac-**7**
Ac-EKNKNANpSSPVASTTPSASATTNPASATTLDQSKAWNR-COOH
MW= 4027.2 Da

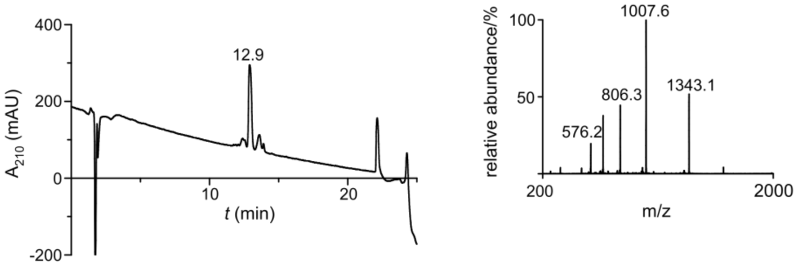

FITC-**7**
FITC-PEG2-EKNKNANpSSPVASTTPSASATTNPASATTLDQSKAWNR-COOH
MW= 4517.0 Da

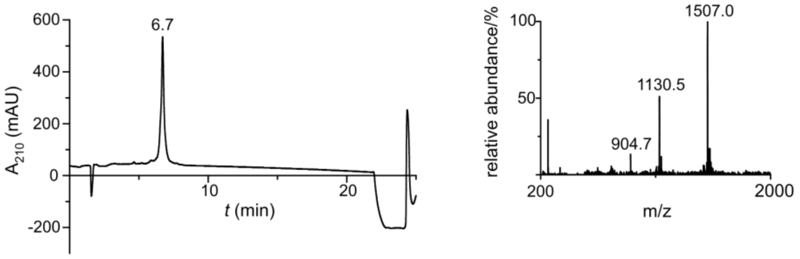

FITC-**8**
FITC-PEG2-EKNKNANpSSPVASTTPSASApTTNPASATTLDQSKAWNR-COOH
MW= 4596.9 Da

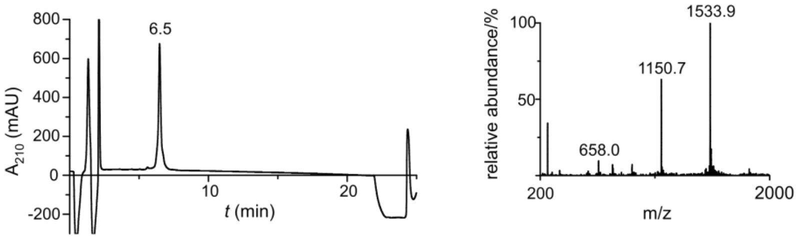

FITC-**9**
FITC-PEG2-EKNKNANpSSPVASTTPSASATpTNPASATTLDQSKAWNR-COOH
MW= 4596.9 Da

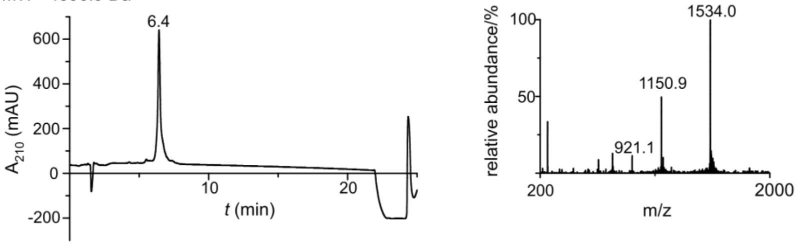

FITC-**10**
FITC-PEG2-EKNKNANpSSPVASTTPSASATTNPApSATTLDQSKAWNR-COOH
MW= 4596.9 Da

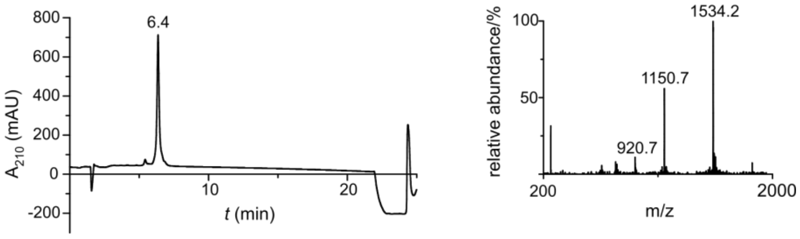

Figure S12. HPLC/ESI-MS analysis. Analysis of peptides Ac-**7**, FITC-**7**, FITC-**8**, FITC-**9** and FITC-**10** performed on Agilent HPLC system using ZORBAX Eclipse XDB-C18 column. Left chromatogram shows Absorption (A_{210}) over time (t) and right diagram show relative abundance of MS-ions.

FITC-**11**
FITC-PEG2-EKNKNANpSSPVASTTPSASATTNPASApTTLDQSKAWNR-COOH
MW= 4596.9 Da

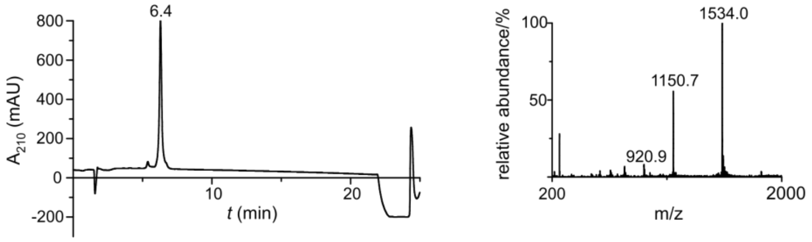

Ac-**11**
Ac-EKNKNANpSSPVASTTPSASATTNPASApTTLDQSKAWNR-COOH
MW= 4104.8 Da

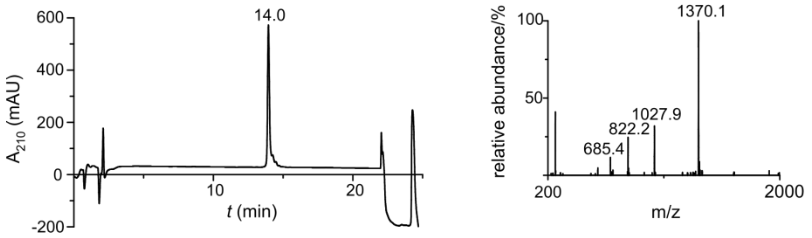

FITC-**12**
FITC-PEG2-EKNKNANpSSPVASTTPSASATTNPASATpTLDQSKAWNR-COOH
MW= 4596.9 Da

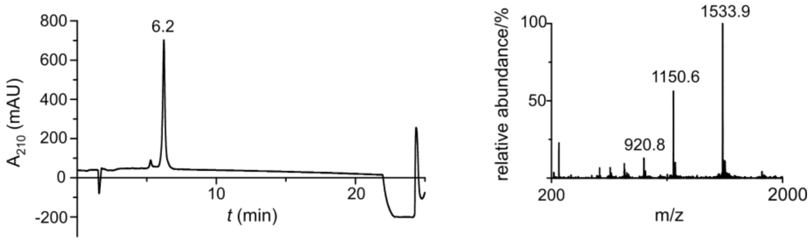

FITC-**13**
FITC-PEG2-EKNKNANpSSPVASTTPSASATTNPASATTLDQpSKAWNR-COOH
MW= 4596.9 Da

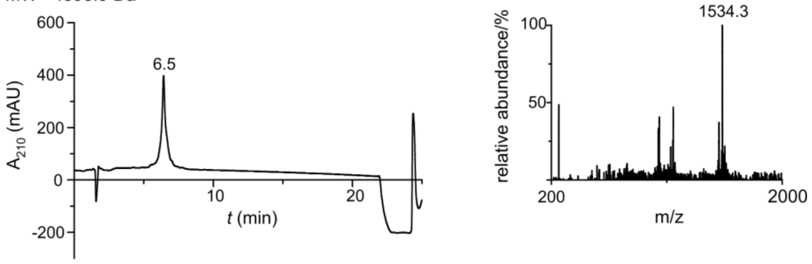

FITC-**14**
FITC-PEG2-TTNPApSATT-COOH
MW= 1476.5 Da

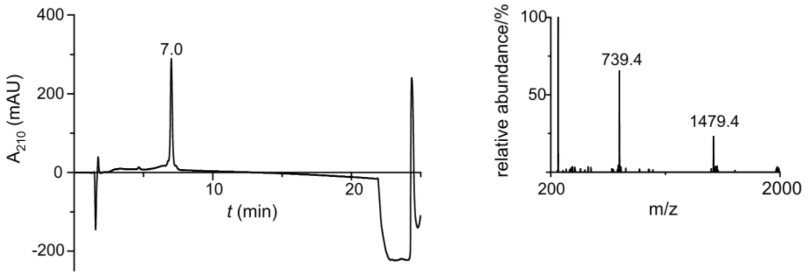

Figure S13. HPLC/ESI-MS analysis. Analysis of peptides FITC-**11**, Ac-**11**, FITC-**12**, FITC-**13** and FITC-**14** performed on Agilent HPLC system using ZORBAX Eclipse XDB-C18 column. Left chromatogram shows Absorption (A_{210}) over time (t) and right diagram show relative abundance of MS-ions.

FITC-**15**
FITC-PEG2-NPASApTTLD-COOH
MW= 1502.5 Da

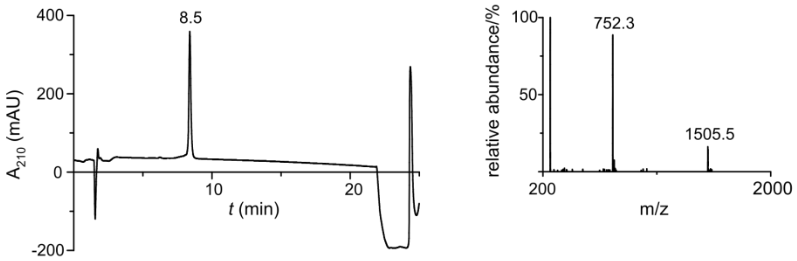

FITC-**16**
FITC-PEG2-PASATpTLDQ-COOH
MW= 1516.5 Da

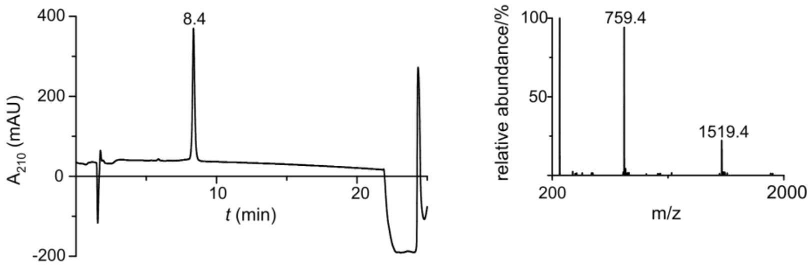

FITC-RAF1
FITC-PEG2-RQRSTpSTPN-COOH
MW= 1659.6 Da

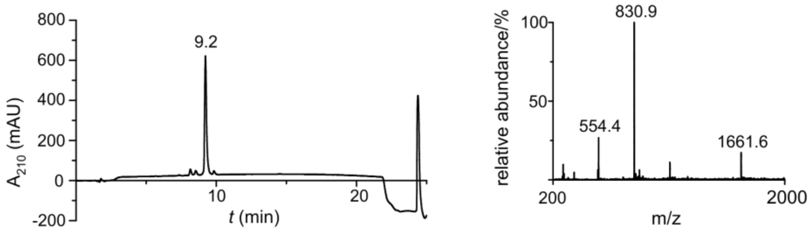

Ac-RAF1
Ac-RQRSTpSTPN-COOH
MW= 1167.5 Da

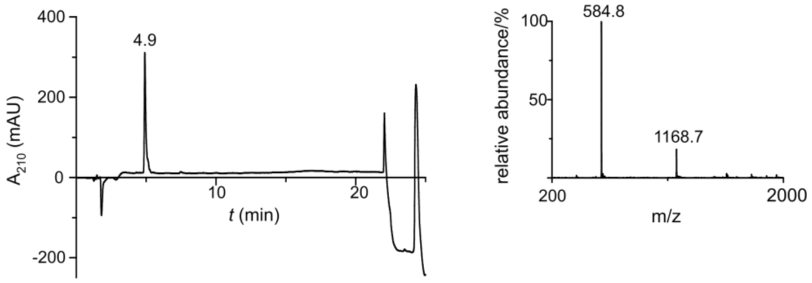

Ac-**17**
Ac-EKNKNANSSPVASTTPSASATTNPASApTTLDQSKAWNR-COOH
MW= 4027.2 Da

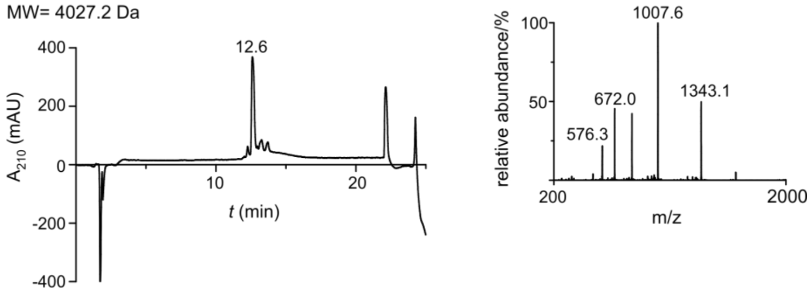

Figure S14. HPLC/ESI-MS analysis**.** Analysis of peptidesFITC-**15**, FITC-**16**, FITC-**RAF1**, Ac-**RAF1** and Ac-**17** performed on Agilent HPLC system using ZORBAX Eclipse XDB-C18 column. Left chromatogram shows Absorption (A_{210}) over time (t) and right diagram show relative abundance of MS-ions.

Table S1. 14-3-3Pred results**.** List of all Ser/Thr residues and corresponding 14-3-3Pred classifiers (ANN, PSSM, SVM). Green highlights indicate matching of threshold. Non-capital letters in sequence indicate potential phosphorylation site. Seven surface exposed residues *italic*. Five final selected peptides in *italic* and **bold**.

aa	peptide	ANN	PSSM	SVM
Ser8	AKGFYIsKSLG	n. d.	n. d.	n. d.
Ser10	GFYISKsLGIL	n. d.	n. d.	n. d.
Thr25	GVAAVCtIIAL	n. d.	n. d.	n. d.
Ser30	CTIIALsVVYS	n. d.	n. d.	n. d.
Ser34	ALSVVYsQEKN	0.134	-0.033	-1.179
Ser43	KNKNANsSPVA	0.626	0.465	0.199
Ser44	NKNANSsPVAS	0.106	-0.322	-1.104
Ser48	NSSPVAsTTPS	0.148	-0.269	-1.185
Thr49	SSPVAStTPSA	0.130	0.167	-0.951
Thr50	SPVASTtPSAS	0.069	-0.262	-1.259
Ser52	VASTTPsASAT	0.083	-0.212	-1.340
Ser54	STTPSAsATTN	0.395	0.110	-0.649
Thr56	TPSASAtTNPA	0.309	0.014	-0.401
Thr57	PSASATtNPAS	0.600	0.445	0.109
Ser61	ATTNPAsATTL	0.237	-0.103	-0.804
Thr63	TNPASAtTLDQ	0.209	-0.026	-0.793
Thr64	NPASATtLDQS	0.391	0.032	-0.186
Ser68	ATTLDQsKAWN	0.145	-0.141	-0.980
Thr79	RYRLPNtLKPD	0.675	0.315	0.164
Ser84	NTLKPDsYRVT	0.165	-0.295	-0.985
Thr88	PDSYRVtLRPY	0.595	0.082	0.041
Thr94	TLRPYLtPNDR	0.346	0.005	-0.348
Ser106	LYVFKGsSTVR	0.128	-0.126	-1.174
Ser107	YVFKGSsTVRF	0.074	-0.206	-1.296
Thr108	VFKGSStVRFT	0.192	-0.030	-0.418
Thr112	SSTVRFtCKEA	0.201	0.074	-0.699
Thr117	FTCKEAtDVII	0.665	0.175	-0.415
Ser124	DVIIIHsKKLN	0.226	0.134	-0.654
Thr130	SKKLNYtLSQG	0.466	0.115	-0.062
Ser132	KLNYTLsQGHR	0.136	-0.024	-0.926
Ser145	LRGVGGsQPPD	0.171	0.392	-0.737
Thr153	PPDIDKtELVE	0.165	-0.011	-0.910
Thr159	TELVEPtEYLV	0.101	-0.123	-1.452
Ser169	VVHLKGsLVKD	0.083	-0.150	-1.204
Ser174	GSLVKDsQYEM	0.101	-0.262	-1.463
Ser180	SQYEMDsEFEG	0.091	-0.168	-1.155
Ser196	LAGFYRsEYME	0.110	-0.105	-1.232
Thr209	VRKVVAtTQMQ	0.219	0.075	-0.868
Thr210	RKVVATtQMQA	0.352	0.034	-0.599
Ser220	AADARKsFPCF	0.759	0.630	0.451
Thr236	KAEFNItLIHP	0.201	-0.140	-0.494
Thr244	IHPKDLtALSN	0.077	-0.160	-1.661
Ser247	KDLTALsNMLP	0.063	-0.244	-1.489
Ser255	MLPKGPsTPLP	0.541	0.447	-0.074
Thr256	LPKGPStPLPE	0.025	-0.624	-1.703
Thr267	DPNWNVtEFHT	0.322	-0.143	-0.562
Thr271	NVTEFHtTPKM	0.402	0.505	-0.231
Thr272	VTEFHTtPKMS	0.097	-0.433	-1.367
Ser276	HTTPKMsTYLL	0.168	0.086	-0.653
Thr277	TTPKMStYLLA	0.079	-0.306	-1.323
Ser285	LLAFIVsEFDY	0.717	0.281	0.404
Ser295	YVEKQAsNGVL	0.307	0.119	-0.597
Ser307	RIWARPsAIAA	0.394	0.342	-0.197
Thr321	DYALNVtGPIL	0.806	0.491	0.383
Thr334	FAGHYDtPYPL	0.059	-0.417	-1.540
Ser341	PYPLPKsDQIG	0.376	0.005	-0.501
Thr361	ENWGLVtYREN	0.110	-0.132	-1.017
Ser366	VTYRENsLLFD	0.677	0.810	0.187
Ser373	LLFDPLsSSSS	0.042	-0.229	-1.511
Ser374	LFDPLSsSSSN	0.025	-0.357	-1.774
Ser375	FDPLSSsSSNK	0.072	-0.072	-1.383
Ser376	DPLSSSsSNKE	0.175	-0.077	-0.937
Ser377	PLSSSSsNKER	0.352	0.112	-0.439
Thr384	NKERVVtVIAH	0.806	0.882	0.766
Thr400	WFGNLVtIEWW	0.088	-0.134	-0.773
Ser415	LNEGFAsYVEY	0.172	-0.126	-0.925
Thr428	ADYAEPtWNLK	0.089	-0.229	-1.205
Ser451	AVDALAsSHPL	0.108	-0.183	-1.292
Ser452	VDALASsHPLS	0.359	0.337	-0.309
Ser456	ASSHPLsTPAS	0.262	0.206	-0.419
Thr457	SSHPLStPASE	0.010	-0.809	-2.624
Ser460	PLSTPAsEINT	0.275	0.051	-0.561
Thr464	PASEINtPAQI	0.104	-0.378	-1.101
Ser469	NTPAQIsELFD	0.098	-0.167	-1.282

aa	peptide	ANN	PSSM	SVM
Ser476	ELFDAIsYSKG	0.207	0.012	-0.830
Ser478	FDAISYsKGAS	0.380	0.354	0.145
Ser482	SYSKGAsVLRM	0.188	-0.158	-1.145
Ser488	SVLRMLsSFLS	0.249	0.644	-0.300
Ser489	VLRMLSsFLSE	0.191	0.074	-0.804
Ser492	MLSSFLsEDVF	0.257	0.115	-0.627
Ser502	FKQGLAsYLHT	0.132	-0.025	-0.887
Thr506	LASYLHtFAYQ	0.162	-0.071	-0.937
Thr512	TFAYQNtIYLN	0.169	0.033	-0.678
Ser529	EAVNNRsIQLP	0.056	-0.205	-1.215
Thr534	RSIQLPtTVRD	0.155	-0.206	-1.123
Thr535	SIQLPTtVRDI	0.313	0.033	-0.437
Thr544	DIMNRWtLQMG	0.591	0.235	-0.026
Thr553	MGFPVItVDTS	0.273	-0.012	-0.570
Thr556	PVITVDtSTGT	0.129	-0.246	-1.287
Ser557	VITVDTsTGTL	0.185	-0.006	-0.689
Thr558	ITVDTStGTLS	0.087	-0.177	-1.341
Thr560	VDTSTGtLSQE	0.060	-0.298	-1.233
Ser562	TSTGTLsQEHF	0.034	-0.291	-1.524
Ser572	FLLDPDsNVTR	0.217	-0.076	-0.944
Thr575	DPDSNVtRPSE	0.420	0.154	-0.594
Ser578	SNVTRPsEFNY	0.252	0.051	-0.916
Thr589	VWIVPItSIRD	0.084	-0.218	-1.145
Ser590	WIVPITsIRDG	0.217	-0.081	-0.394
Ser613	AQNDLFsTSGN	0.070	-0.210	-1.423
Thr614	QNDLFStSGNE	0.072	-0.255	-1.545
Ser615	NDLFSTsGNEW	0.302	0.132	-0.240
Thr627	LLNLNVtGYYR	0.322	-0.026	-0.554
Thr644	NWRKIQtQLQR	0.459	0.229	-0.233
Ser651	QLQRDHsAIPV	0.735	1.055	0.371
Ser670	DAFNLAsAHKV	0.179	-0.066	-1.391
Thr677	AHKVPVtLALN	0.266	-0.072	-0.765
Thr683	TLALNNtLFLI	0.678	0.266	0.333
Ser700	PWEAALsSLSY	0.111	-0.074	-1.125
Ser701	WEAALSsLSYF	0.070	-0.271	-1.203
Ser703	AALSSLsYFKL	0.133	-0.031	-0.847
Ser712	KLMFDRsEVYG	0.209	0.086	-0.915
Thr727	YLKKQVtPLFI	0.275	-0.176	-0.622
Thr737	IHFRNNtNNWR	0.768	0.908	0.412
Ser752	NLMDQYsEVNA	0.262	0.276	-0.593
Ser758	SEVNAIsTACS	0.084	-0.113	-1.307
Thr759	EVNAIStACSN	0.035	-0.250	-1.607
Ser762	AISTACsNGVP	0.319	0.017	-0.937
Ser773	ECEEMVsGLFK	0.096	-0.297	-1.260
Ser794	IHPNLRsTVYC	0.036	-0.317	-2.071
Thr795	HPNLRStVYCN	0.160	0.142	-0.945
Thr820	EQFRNAtLVNE	0.731	0.965	0.620
Ser835	RAALACsKELW	0.307	0.063	-0.703
Ser846	ILNRYLsYTLN	0.671	0.864	0.266
Thr848	NRYLSYtLNPD	0.654	0.586	0.613
Thr860	IRKQDAtSTII	0.606	0.224	0.013
Ser861	RKQDATsTIIS	0.463	0.177	-0.353
Thr862	KQDATStIISI	0.033	-0.230	-1.219
Ser865	ATSTIIsITNN	0.209	-0.149	-1.043
Thr867	STIISItNNVI	0.383	0.212	-0.447
Ser882	VWDFVQsNWKK	0.178	-0.104	-1.146
Ser895	NDYGGGsFSFS	0.023	-0.376	-1.750
Ser897	YGGGSFsFSNL	0.253	0.285	-0.247
Ser899	GGSFSFsNLIQ	0.327	0.124	-0.586
Thr906	NLIQAVtRRFS	0.376	-0.065	-0.529
Ser910	AVTRRFsTEYE	0.643	0.914	0.303
Thr911	VTRRFStEYEL	0.783	1.092	0.501
Thr928	KKDNEEtGFGS	0.124	-0.236	-1.102
Ser932	EETGFGsGTRA	0.084	-0.342	-1.455
Thr934	TGFGSGtRALE	0.140	-0.099	-0.974
Thr944	EQALEKtKANI	0.201	-0.011	-0.545
Thr963	VVLQWFtENSK	0.200	0.034	-0.383
Ser966	QWFTENsK---	0.247	0.130	-0.907
